# HBx hijacks the miR‐19a‐3p/BAMBI/TGF‐β1 axis to impair the anti‐tumour activity of CD4^+^ T cells in diffuse large B‐cell lymphoma

**DOI:** 10.1002/ctm2.70578

**Published:** 2026-01-05

**Authors:** Xuecong Guo, Jianguo Li, Xiaofei Bai, Yinghui Huang, Xu Xu, Jiabang Yang, Zhenghao Sun, Wangcheng Zhu, Xudong Guo, Jie Chen, Jiuhong Kang

**Affiliations:** ^1^ Clinical and Translational Research Center of Shanghai First Maternity and Infant Hospital, Shanghai Key Laboratory of Maternal Fetal Medicine, Shanghai Key Laboratory of Signaling and Disease Research, Frontier Science Center for Stem Cell Research, National Stem Cell Translational Resource Center, School of Life Sciences and Technology Tongji University Shanghai China; ^2^ Department of Hematology Changhai Hospital Naval Medical University Shanghai China; ^3^ Department of Life Sciences Imperial College London London UK

**Keywords:** CD4+ T cells, diffuse large B‐cell lymphoma (DLBCL), hepatitis B virus (HBV), prognosis

## Abstract

**Background:**

Hepatitis B virus (HBV) is clinically associated with poor prognosis in diffuse large B‐cell lymphoma (DLBCL), while cellular communication in the tumour microenvironment (TME) is recognized as a critical driver of tumour progression. Nevertheless, whether HBV infection mediates DLBCL cell‐immune cell crosstalk remains undefined, with the precise mechanisms and associated key molecules remaining elusive.

**Methods:**

SsGSEA, Cox regression (univariate/multivariate), WGCNA, and Kaplan–Meier analyses identified prognostic immune subsets and miRNAs in HBV^+^ DLBCL. Dual luciferase assay, qRT‐PCR, western blot, ChIP, Co‐IP, flow cytometry, enzyme‐linked immunosorbent assay, immunohistochemistry, and murine models were employed together to evaluate CD4^+^ T cell dysfunction in vitro and in vivo. ScRNA‐seq analyses encompassed clustering, pseudotemporal trajectory, and ligand–receptor networks to decode TME dynamics.

**Results:**

TME profiling identified diminished CD4⁺ T cell infiltration as an independent predictor of poor survival in HBV⁺ DLBCL. Mechanistically, HBx‐mediated down‐regulation of miR‐19a‐3p activated the BAMBI/Wnt signalling pathway, thereby enhancing TGF‐β1 secretion and suppressing the anti‐tumour activity of CD4^+^ T cells. Single‐cell analysis revealed that BAMBI^high^ DLBCL cells engage CD4^+^ T cells via TGFB1‐TGFBR2 pair, with TGFBR2 enriched in exhausted subsets of CD4^+^ T cells and shaping their dysfunctional fate. Therapeutic restoration of miR‐19a‐3p or blockade of TGF‐β reinforced the CD4⁺ T cell anti‐tumour activity and restrained the progression of HBx‐overexpressing DLBCL in vivo.

**Conclusions:**

HBx promoted TGF‐β1 hypersecretion via miR‐19a‐3p repression‐mediated Wnt/β‐catenin activation, directly driving CD4^+^ T cell depletion and functional exhaustion in DLBCL. Our work provided important insights into the immune determinants of poor prognosis in HBV^+^ DLBCL, highlighting the pivotal role of CD4^+^ T cell dysfunction in driving disease progression and adverse clinical outcomes.

**Highlights:**

1. Reduced CD4^+^ T cell enrichment in the TME predicted poor survival in HBV^+^ DLBCL.
2. Down‐regulation of miR‐19a‐3p by HBx activated the BAMBI‐mediated Wnt signalling, amplifying TGF‐β1 secretion to suppress anti‐tumour activity of CD4⁺ T cells.
3. The TGF‐B1/TGFBR2 pair mediated the HBV^+^ DLBCL‐CD4^+^ T cell communication.
4. Targeting TGF‐β or miR‐19a‐3p improved CD4^+^ T cell immunity to suppress HBV^+^ DLBCL progression

## Graphical abstract

HBx down‐regulated miR‐19a‐3p, leading to activation of BAMBI‐mediated Wnt signalling and increased TGF‐β1 secretion by malignant B cells. The TGF‐B1/TGFBR2 signalling axis mediated tumour‐CD4^+^ T cell communication, suppressing CD4^+^ T cell anti‐tumour activity and resulting in reduced CD4^+^ T cell enrichment and poor prognosis. Therapeutic targeting of TGF‐β or restoration of miR‐19a‐3p enhanced CD4^+^ T cell immunity and limited HBV^+^ DLBCL progression.

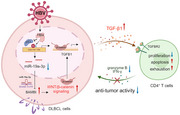

## 1 INTRODUCTION

Diffuse large B‐cell lymphoma (DLBCL) is the predominant subtype of non‐Hodgkin lymphoma, with nearly 40% of cases occurring in extranodal sites, characterized by its highly aggressive nature.^1^ HBV coinfection is prevalent in DLBCL populations, with affected patients exhibiting younger onset age, advanced‐stage disease, suboptimal response to frontline immunochemotherapy, and reduced survival.^2^ Accumulating data indicate that HBV infection is potentially involved in the onset of DLBCL through the induction of oncogenic pathways and alterations in the tumour microenvironment (TME). HBV can activate NF‐κB signalling, thereby promoting anti‐apoptotic programs including BCL‐2‐mediated survival.^3^ These alterations, together with MYC rearrangements and aberrant BCL‐6 regulation,^4^, ^5^ as well as epigenetic dysregulation,^6^, ^7^ drive B‐cell transformation and clonal expansion. In parallel, mutations in immune‐related genes such as CD70, TNFRSF14, and CD58 weaken T‐cell‐mediated immune surveillance,^8^ while disruption of apoptosis (Caspase‐3‐PARP) and cell‐cycle control further accelerates lymphoma progression and therapeutic resistance.^9^, ^10^ Accumulating studies have highlighted TME as a critical determinant of tumour progression and clinical outcomes.^11^ Diverse immune and stromal elements, encompassing tumour‐infiltrating lymphocytes, macrophages, fibroblasts, and signalling mediators such as cytokines and chemokines, collectively contribute to the ecological diversity of DLBCL and are associated with disease heterogeneity and prognosis.^12^, ^13^ However, how HBV^+^ DLBCL remodels a distinct TME landscape remains poorly understood.

Immune evasion driven by communication between tumour cells and immune cells in the TME contributes to adverse patient outcomes. Breast cancer cells compromise antigen presentation by diminishing MHC‐I surface expression and stability.^14^ M1 macrophages within the gastric cancer TME displayed cytotoxic effects on tumour cells and boosted effector activity via stimulation of key cytotoxic lymphocytes, including CD8^+^ T cells and NK cells.^15^ Recent studies have confirmed that HBV is a major factor in regulating cell communication within the TME. Exosomes secreted by HBV‐infected hepatocellular carcinoma (HCC) cells have been reported to shuttle into NK cells, thereby impairing their function, proliferation, and survival.^16^ HBV further co‐opts immune evasion by up‐regulating PD‐L1 on HCC cells, which engages PD‐1^+^ CD8^+^ T cells to dysregulate anti‐tumour immunity and accelerate hepatocarcinogenesis.^17^, ^18^, ^19^ The impact of HBV infection on DLBCL‐TME communication dynamics and its mechanistic basis remains unexplored.

MicroRNAs (miRNAs) are emerging as versatile therapeutic agents due to their capacity for precise targeting via synthetic mimics or inhibitors.^20^, ^21^ These non‐coding RNAs orchestrate both cell‐intrinsic processes (proliferation/apoptosis) and intercellular crosstalk within the TME, positioning them as dual regulators of tumour biology and immunotherapy response. MiR‐34a in breast cancer cells manipulates IL‐6/IL‐6R signalling to drive macrophages towards a pro‐tumour M2 phenotype, thereby facilitating cancer progression.^22^ Similarly, exosomes from ovarian tumours carrying miR‐155‐5p diminish PD‐L1 levels on macrophages, enhancing cytotoxic T lymphocyte activity.^23^ In DLBCL cells, miR‐155 targets the AKT/ERK signalling pathway to down‐regulate PD‐L1 expression,^24^ while miR‐340‐5p regulates the ubiquitination of CD73,^25^ both of which modulate the infiltration and anti‐tumour activity of CD8^+^ T cells. HBV hijacks miRNA networks in HCC (miR‐181a, miR‐21, etc.) to subvert immune surveillance by dysregulating inflammatory mediators (TNF‐α, IFN‐γ, etc.) and signalling hubs (NOTCH, TCR signalling, etc.).^26^, ^27^, ^28^ However, it remains unknown whether HBV‐responsive miRNAs modulate immune crosstalk in DLBCL and underlie its dismal prognosis.

Our study revealed that HBV core protein HBx suppressed miR‐19a‐3p, a critical regulator restoring CD4^+^ T cell anti‐tumour activity in the DLBCL microenvironment. Mechanistically, miR‐19a‐3p directly silenced BAMBI, a Wnt/β‐catenin amplifier, triggering TGF‐β1 hypersecretion in HBx‐expressing DLBCLs. BAMBI^high^ DLBCL cells suppressed CD4^+^ T cell function via the TGFB1‐TGFBR2 pair; TGFBR2 was preferentially expressed in exhausted CD4^+^ T cells. Notably, both miR‐19a‐3p overexpression and TGF‐β antibody antagonism may serve as promising therapeutic strategies to reverse CD4^+^ T cell suppression and ameliorated the prognosis of HBV‐related DLBCL.

## 2 MATERIALS AND METHODS

### 2.1 Cell culture

Human SUDHL‐4 and HEK293T cells (ATCC, USA) and murine EL4 B‐lymphoma cells (Shanghai Fuheng Biotechnology) were authenticated by STR profiling and routinely screened for mycoplasma. SUDHL‐4 cells were maintained in RPMI 1640, while HEK293T and EL4 cells were cultured in DMEM, each supplemented with 10% FBS (Gibco, USA) at 37°C with 5% CO_2_. CD4^+^ T cells were purified using magnetic‐activated CD4 microbeads (Miltenyi Biotec, Germany) from human PBMCs (Milecell Biotechnologies, P123040210C). CD4^+^ T cells were subsequently cultured in X‐VIVO medium (Lonza, Switzerland) containing 20 IU/mL IL‐2 (MedChemExpress, USA) and activated with T Cell TransAct (Miltenyi Biotec).

### 2.2 Public data collection and processing

The miRNA‐seq and RNA‐seq data were sourced from the TCGA‐DLBC cohort (http://cancergenome.nih.gov/), comprising 48 RNA‐seq and 47 miRNA‐seq profiles from DLBCL patients. After filtering out outliers and samples with missing clinical data, 46 DLBCL samples were retained for analysis. Bulk RNA‐seq datasets (GSE10846, *n* = 420, with 250 samples retained for survival analysis; GSE23501, *n* = 69, with 43 R‐CHOP‐treated patients included; GSE178965, *n* = 19; GSE125966, *n* = 553; GSE87371, *n* = 223) and scRNA‐seq data (GSE182434) with clinical annotations were sourced from GEO repository (https://www.ncbi.nlm.nih.gov/geo/). For bulk datasets, the normalized expression matrices provided by the original studies were used. For GSE182434, low‐quality cells were excluded based on gene number (<200 or >6000) and mitochondrial gene content (>10%).

Single‐sample gene set enrichment analysis (ssGSEA) with the MM3 metagenes matrix (782 genes covering 28 hematopoietic cell types) was used to assess immune cell infiltration from RNA‐seq data. Samples were classified by immune cell scores for Cox regression and survival analysis, which also included miRNA and gene expression. Weighted gene co‐expression network analysis (WGCNA) was applied to the TCGA‐DLBC cohort. For scRNA‐seq data, CD4^+^ T cells underwent cluster analysis (Seurat v5.2.1) and developmental trajectory mapping (Monocle2). Malignant B cells were discriminated from normal infiltrating B cells based on CNV scores (InferCNV), followed by GSVA analysis of malignant populations. Cell‐cell interactions were analyzed using the CellChat package.

### 2.3 Microarray analysis of miRNAs

SUDHL‐4 cell RNA was purified using RNAiso reagent (Takara, Japan), and the RNA Integrity Number was checked using Agilent Bioanalyzer 2100 (Agilent Technologies, USA). Subsequently, Cyanine‐5‐labelled amino‐allyl antisense RNA was ligated to NHS‐CyDye, purified, and quantified via NanoDrop ND‐1000 spectrophotometer. Annotation was based on the miRBase database release 21. To ensure reliable data, miRNAs with detection *p*‐values exceeding  .05 in either control or HBx‐overexpressing samples were filtered out before subsequent analyses. Normalized miRNA array data are provided in Table S1.

### 2.4 Transfection and genome editing

SUDHL‐4 cells received miR‐19a‐3p mimics, inhibitor, or control (Riobio, China) via Lipofectamine 2000 (Invitrogen, USA), while HEK293T cells were engineered with HBx/miR‐19a‐3p overexpression vectors or BAMBI/TGFBR2 shRNA constructs using identical transfection protocols. Lentiviral particles (∼1 × 10⁸ TU/mL) were applied to SUDHL‐4, EL4, and CD4^+^ T cells, followed by puromycin selection.

To generate gene‐edited cell lines, SUDHL‐4 cells were engineered using plasmid‐based CRISPR systems delivered by electroporation. For selective suppression of miR‐19a‐3p at the RNA level, CRISPR/Cas13d was employed to specifically target mature miR‐19a‐3p transcripts. In contrast, BAMBI and TGFB1 were genetically ablated using a CRISPR/Cas9‐based approach. Guide RNA details are summarized in Table S2. Electroporation was conducted in accordance with the manufacturer's guidelines, followed by a 48 h recovery. Single‐cell‐derived clones were obtained by limiting dilution and expanded for 2 weeks.

### 2.5 Luciferase report assay

The predicted BAMBI 3′UTR sequences bound by miR‐19a‐3p and p53 binding sites at the *hsa‐mir‐19a* promoter were cloned into the pGL3‐basic vector. HEK293T cells were co‐transfected with miRNA mimics/inhibitors or plasmids along with luciferase reporters; dual‐luciferase activities (Firefly/Renilla) were quantified at 48 h using Promega's detection system.

### 2.6 ELISA

Levels of human IFN‐γ, Granzyme B, and TGF‐β1 were measured using human‐specific ELISA kits (Absin, # abs510007; R&D Systems, # DGZB00; Jianglai Bio, # JL10706) and mouse‐specific ELISA kits for syngeneic models (Absin, # abs520007; Jianglai Bio, # JL11913).

### 2.7 Cell apoptosis assay

Cell apoptosis was quantified using Annexin V‐FITC/PI dual staining (KeyGen BioTECH, China). After 10‐minute dark incubation, samples were analyzed on a BD flow cytometer, with data processed with FlowJo (Tree Star, USA).

### 2.8 CFSE dilution assay

CD4^+^ T cells (1 × 10^6^ cells/mL) underwent CFSE labelling by incubation with 2.5 µmol/L working solution (prepared from 10 mmol/L DMSO stock) for 15 min at 37°C. After quenching with complete medium, cells were cultured in 24‐well plates under specified conditions. Proliferation was analyzed using a BD Accuri C6 Plus flow cytometer and FlowJo software.

### 2.9 ChIP‐qPCR

ChIP was performed according to standard procedures.^10^ Antibodies included anti‐p53 (Abcam, # ab26, RRID: AB_303198), anti‐H3K27ac (Abcam, # ab4729 RRID: AB_2118291), Normal Mouse IgG Polyclonal Antibody (Millipore, # 12–371 RRID: AB_145840), and Normal Rabbit IgG Polyclonal Antibody (Cell Signaling Technology, # 2729 RRID: AB_1031062). DNA isolation was performed via phenol‐chloroform, followed by qPCR targeting the *hsa‐mir‐19a* promoter (Table S2). Values were normalized to the input control.

### 2.10 Co‐immunoprecipitation

SUDHL‐4 cells were lysed in IP buffer with protease inhibitors and sonicated. Lysates were pre‐cleared by incubation with Protein A/G beads to remove nonspecific proteins, with a portion kept as input. The remaining lysate was incubated with beads pre‐bound to 3 µg of anti‐HBx (Thermo Fisher Scientific, # MA1081, RRID: AB_325418), anti‐p53 (Abcam, #ab26, RRID: AB_303198), or isotype control IgG for 6 h at 4°C. Proteins captured on the beads were eluted using SDS buffer and subjected to western blot analysis.

### 2.11 Western blot

Western blot analysis was carried out using conventional methods.^29^ Antibodies included: anti‐HBx, anti‐p53, anti‐BAMBI (Thermo Fisher Scientific, # PA5‐38027, RRID: AB_2554631), anti‐β‐catenin (Abcam, # ab16051, RRID: AB_443301), anti‐GAPDH (Bioworld, # AP0063, RRID: AB_2651132), HRP‐Rabbit (Cell Signaling Technology, # 7074, RRID: AB_2099233), and HRP‐Mouse (Cell Signaling Technology, # 7076, RRID: AB_330924).

### 2.12 Co‐culture system

Transwell chambers (.4 µm, Sarstedt, Germany) were utilized for the co‐culture assay. CD4^+^ T cells (2 × 10^5^) and SUDHL‐4 cells (1 × 10^5^) were introduced into the upper and lower chambers, respectively, for 72 h.

### 2.13 Immunohistochemical staining

Paraffin‐embedded tumour sections (DLBCL and syngeneic) were prepared and subjected to antigen retrieval, followed by incubation with primary antibodies overnight and HRP‐conjugated secondary antibodies, according to standard protocols. Antibodies included anti‐CD4 (Abcam, # ab237722, RRID: AB_3431924), anti‐HBx, anti‐BAMBI, anti‐TGF‐β1 (Servicebio, # GB11179‐100, RRID: AB_ 3661656), anti‐HBsAg (Absin, # abs171569‐100), and HRP‐conjugated goat anti‐rabbit IgG (Servicebio, # GB23303, RRID: AB_2811189).

### 2.14 Murine model

Tumours were established in 8‐week‐old female C57BL/6N mice (Beijing Vital River Laboratory Animal Center) by injecting 1×10^6^ EL4 cells into the right groin flank. Once tumours reached  .5 cm in diameter, treatment was started. MiR‐19a‐3p agomir or agomir NC (Riobio) was administered intratumourally (5 nmol/dose) every 3 days. Anti‐TGF‐β antibody (Selleck, #A2113; 200 µg/dose) or the combination of anti‐PD‐1 (Selleck, #A2122; 200 µg/dose) and anti‐CTLA‐4 antibody (Selleck, #A2103; 200 µg/dose) was delivered intravenously every 3 days.

For the humanized CD4^+^ T cell adoptive transfer model, 8‐week‐old female NOD/SCID mice (Beijing Vital River Laboratory Animal Center) were subcutaneously inoculated in the right flank with 1 × 10^7^ SUDHL‐4 cells mixed with Matrigel at a 1:2 ratio. After tumour establishment, purified human CD4^+^ T cells (1 × 10^6^ cells/mouse) were introduced once via tail‐vein injection.^30^ Concurrently, miR‐19a‐3p agomir or agomir NC was delivered intratumourally every 3 days throughout tumour progression. Tumour volumes were calculated with the formula  .5 × length × width^2^.

### 2.15 Statistical analysis

Statistical analyses were conducted with GraphPad Prism 7.0 (GraphPad Software, USA) and R v4.0.2. Continuous variables are presented as means ± SD from at least three biological replicates, assessed by Student's *t*‐test, ANOVA (one/two‐way), or Pearson correlation. Clinical outcome analyses employed Cox proportional hazards regression and Kaplan–Meier survival curves.

## 3 RESULTS

### 3.1 CD4^+^ T cell depletion is an independent prognostic marker in HBV^+^ DLBCL

**FIGURE 1:**
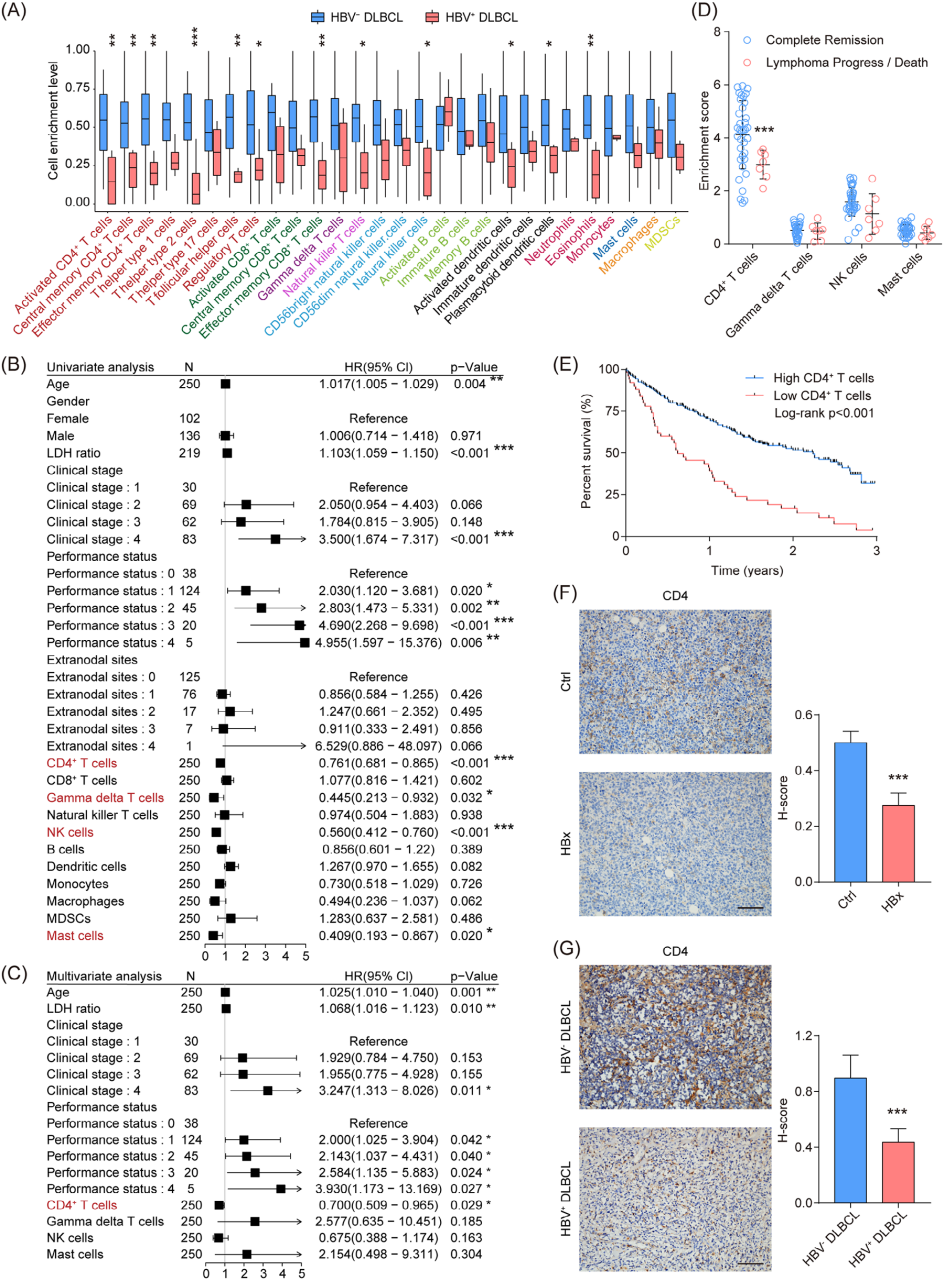
CD4^+^ T cell depletion is an independent prognostic marker in HBV^+^ DLBCL. (A) A box plot showing the enrichment fractions of immune cell subsets in the TME of DLBCL patients (4 HBV^+^ and 42 HBV^−^ DLBCL cases, TCGA‐DLBC). (B, C) Univariate (B) and multivariate (C) regression analyses of the relationship between immune cell infiltration fractions and DLBCL prognosis (GSE10846). (D) Patients with poor response to R‐CHOP treatment exhibited lower enrichment scores of CD4^+^ T cells (GSE23501). (E) Patients with low enrichment scores of CD4^+^ T cells had shorter survival time (GSE10846). (F, G) IHC analysis of CD4 in mouse lymphoma tissues (F) and DLBCL tumours (G). Scale bar = 100 µm. The error bars represented the mean ± SD. Statistical significance was determined by comparison with the corresponding control group unless otherwise indicated. *p < .05, **p < .01, ***p < .001.

To evaluate the enrichment levels of immune cells and their prognostic relevance in HBV^+^ DLBCL tissues, we first assessed the immunological features of 46 tumour samples from the TCGA‐DLBC cohort, using immune‐related gene sets and the ssGSEA algorithm. Compared with HBV^−^ DLBCL patients, HBV^+^ DLBCL patients exhibited reduced enrichment of various immune cells in the TME, such as activated CD4^+^ T cells, Th2 cells, effector memory CD8^+^ T cells, and eosinophils (Figure 1A). Validation in the GSE10846 cohort (*n* = 250) identified CD4^+^ T cells, γδ T cells, NK cells, and mast cells as prognostically significant via univariate regression (Figure 1B). Further multivariate analysis confirmed CD4^+^ T cells as independent prognostic factors for DLBCL patients (Figure 1C). Additionally, DLBCL patients with tumour progression or death showed diminished CD4^+^ T cell enrichment in the TME compared with complete remission cases (Figure 1D; Figure S1A). A higher international prognostic index (IPI) was correlated with reduced CD4^+^ T cell abundance in DLBCL tissues (Figure S1B). Lower CD4^+^ T cell enrichment was associated with a markedly reduced 3‐year survival among DLBCL patients (Figure 1E). The genomic PCR analysis confirmed HBV genes (X, S, P, and C) were integrated into HBV^+^ DLBCL tissues (Figure S2A), and immunohistochemistry further revealed the HBx protein was expressed within tumour cells and HBsAg was primarily located in tumour interstitial regions (Figure S2B,C). Consistently, reduced CD4^+^ T cell enrichment was observed in HBx‐overexpressing murine models and HBV^+^ DLBCL patient tissues (Figure 1F,G). These findings underscored CD4^+^ T cell enrichment in the TME as a critical prognostic determinant in HBV^+^ DLBCL.

### 3.2 The CD4^+^ T cell enrichment‐associated miR‐19a‐3p is suppressed by the HBV core protein HBx

**FIGURE 2:**
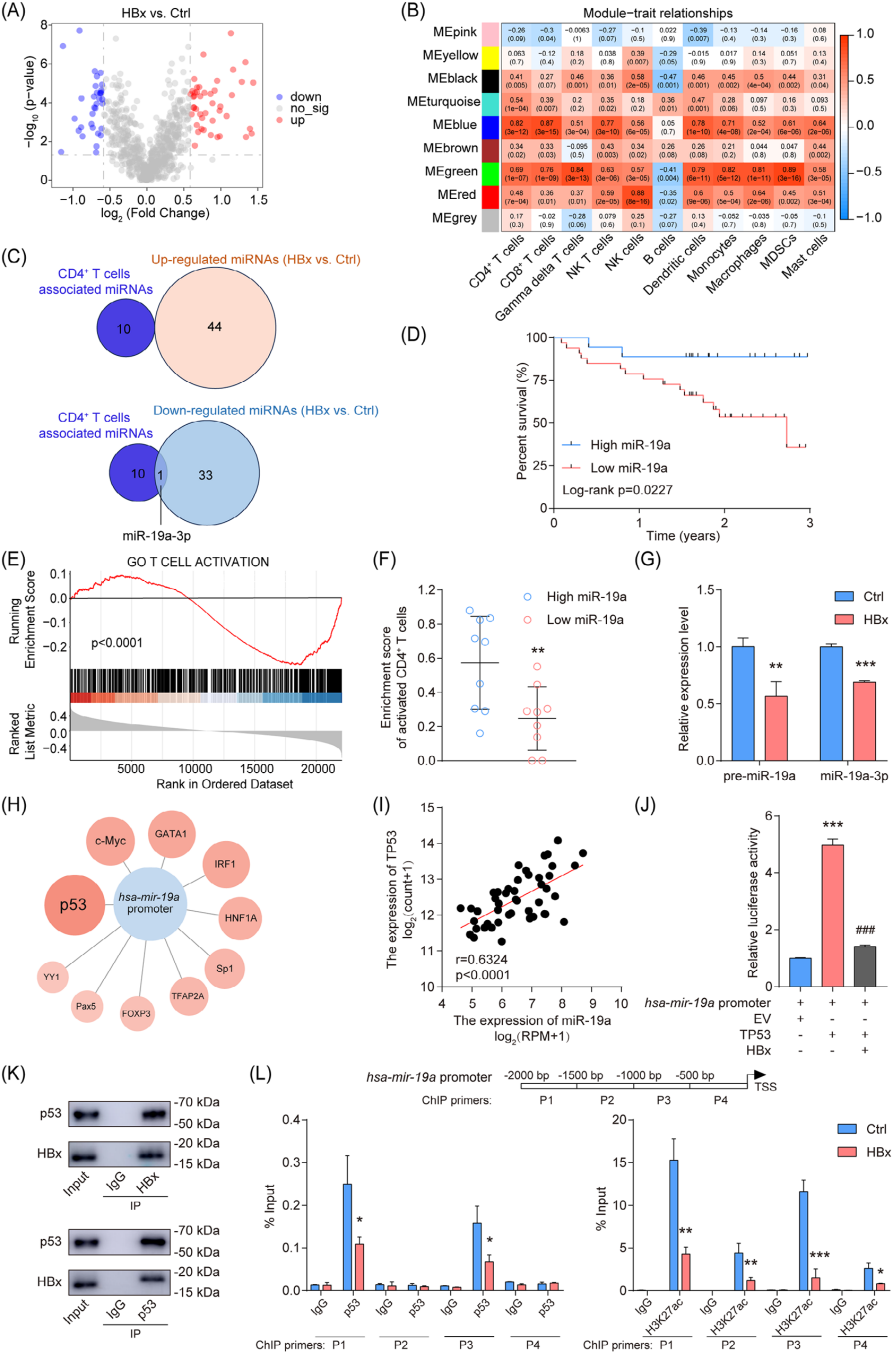
The CD4^+^ T cell enrichment‐associated miR‐19a‐3p is suppressed by the HBV core protein HBx. (A) A volcano plot displaying differentially expressed miRNAs (fold change ≥ 1.5, *p*‐value ≤  .05) in HBx‐overexpressing DLBCL cells compared with control cells. (B) A heatmap showing the correlation between nine modules and eleven types of immune cells (TCGA‐DLBC). (C) Intersection of hub miRNAs identified in the WGCNA and differentially expressed miRNAs. (D) Survival analysis of miR‐19a‐3p in DLBCL patients (TCGA‐DLBC). (E) GSEA indicated genes associated with miR‐19a‐3p were related to T cell activation (TCGA‐DLBC). (F) The low expression group of miR‐19a exhibited a lower enrichment score of CD4^+^ T cells compared with the high expression group (TCGA‐DLBC). (G) The expression of pre‐miR‐19a and miR‐19a‐3p after HBx overexpression was analyzed by qRT‐PCR. (H) Prediction of transcription factors binding at the *hsa‐mir‐19a* promoter region. (I) A significant correlation was observed between miR‐19a and TP53 in DLBCL tissues (TCGA‐DLBC). (J) The effect of TP53 and HBx on the transcriptional activity of the *hsa‐mir‐19a* promoter was assessed using a luciferase reporter assay in HEK293T cells. (K) Co‐immunoprecipitation of HBx and p53 in HBx‐expressing DLBCLs. (L) ChIP‐qPCR of p53 and H3K27ac at the *hsa‐mir‐19a* promoter. The data represent mean ± SD of at least three independent experiments. *p < .05, **p < .01, and ***/^###^ *p* < .001.

To identify miRNAs associated with the reduced CD4^+^ T cell enrichment observed in HBV^+^ DLBCL, we analyzed the miRNA microarray data from HBx‐overexpressing SUDHL‐4 cells, identifying 78 differentially expressed miRNAs (44 up‐regulated, 34 down‐regulated), including HBx‐regulated miRNAs such as miR‐19a‐3p, miR‐486‐5p, and miR‐3960 (Figure 2A). WGCNA of gene‐miRNA expression profiles identified a blue module strongly associated with CD4^+^ T cells (*r* = .82, *p* = 3e^−12^), which harboured 11 miRNAs (e.g., miR‐19a‐3p, miR‐196b, miR‐182) (Figure 2B). Notably, miR‐19a‐3p exhibited dual characteristics: CD4^+^ T cell association and HBx‐induced suppression (Figure 2C). In the TCGA‐DLBC cohort, low miR‐19a‐3p expression predicted poor 3‐year survival by Kaplan–Meier analysis (Figure 2D). Functional annotation of miR‐19a‐3p‐associated transcripts using GSEA revealed significant enrichment in T cell activation pathways, indicating its regulatory role in T cell immunity (Figure 2E). Consistently, samples with low miR‐19a‐3p expression exhibited markedly reduced CD4^+^ T cell activation relative to samples with high expression (Figure 2F).

To further clarify the mechanism underlying HBx‐mediated suppression of miR‐19a‐3p, we observed that HBx overexpression coordinately reduced both mature miR‐19a‐3p and its precursor (pre‐miR‐19a) levels (Figure 2G). Given that HBx regulates transcription indirectly through protein interactions rather than direct DNA binding,^31^ we screened nine transcription factors (predicted by PROMO and JASPAR databases) for potential binding to the *hsa‐mir‐19a* promoter, identifying p53 as the top candidate (Figure 2H). In the TCGA‐DLBC cohort, TP53 expression was positively associated with miR‐19a levels (Figure 2I). Dual‐luciferase reporter assays confirmed direct p53 binding to the *hsa‐mir‐19a* promoter, with HBx co‐expression significantly attenuating p53‐induced luciferase activity (Figure 2J). Endogenous co‐immunoprecipitation further confirmed a physical interaction between HBx and p53 (Figure 2K). Consistent with reports that HBx impairs p53‐mediated transcriptional complex recruitment,^32^ HBx expression significantly reduced p53 occupancy at the promoter region (−2.0 to −1.5 kb and −1.0 to −.5 kb positions) (Figure 2L). ChIP assay for H3K27ac demonstrated a pronounced reduction in histone acetylation across the entire *hsa* *‐mir‐19a* promoter in HBx‐expressing SUDHL‐4 cells, with marked decreases particularly at −2.0 to −1.5 kb and −1.0 to −.5 kb positions (Figure 2L). Together, these findings suggested that HBx interacts with p53 and attenuates its binding to the *hsa* *‐mir‐19a* promoter, along with the reduction of histone acetylation and down‐regulation of miR‐19a‐3p expression.

### 3.3 MiR‐19a‐3p promotes the apoptosis of HBx‐expressing DLBCLs by enhancing CD4^+^ T cells’ anti‐tumour activity

**FIGURE 3:**
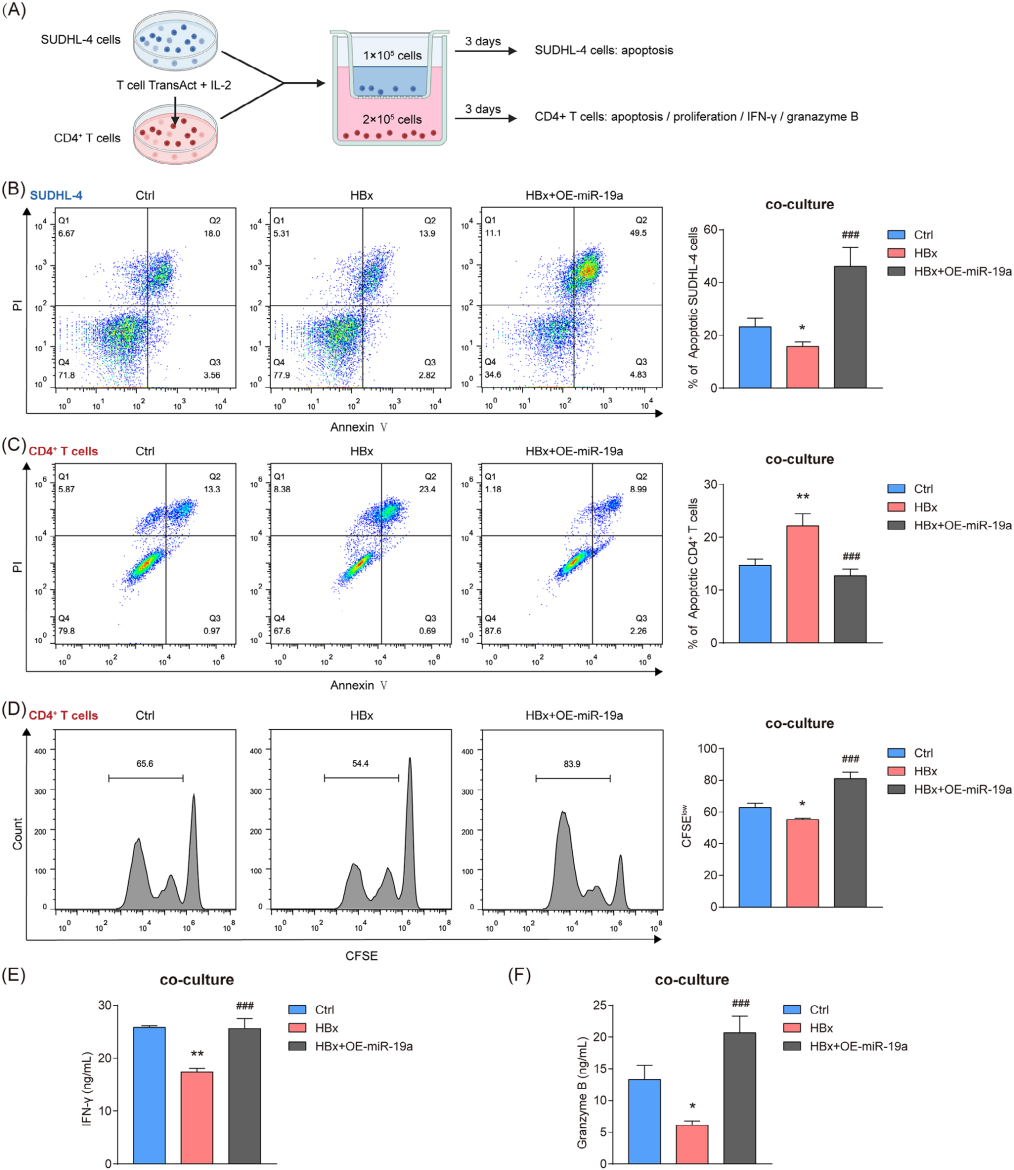
MiR‐19a‐3p promotes the apoptosis of HBx‐expressing DLBCLs by enhancing CD4^+^ T cells’ anti‐tumour activity. (A) Co‐culture model. (B) Flow cytometry analysis for the changes in the proportion of apoptotic SUDHL‐4 cells within the co‐culture system. (C, D) Flow cytometry analysis for the changes in the proportions of apoptotic (C) and proliferative (D) CD4^+^ T cells in the co‐culture system, assessed by Annexin V‐FITC/PI staining and CFSE assay, respectively. (E, F) The levels of IFN‐γ (E) and Granzyme B (F) in the supernatant were measured by ELISA. The data represent mean ± SD of three independent experiments. *p < .05, **/^##^ *p* < .01, ***/^###^ *p* < .001.

To investigate the potential role of miR‐19a‐3p in DLBCL cells, we performed CCK‐8 and apoptosis assays, which revealed that neither HBx nor miR‐19a‐3p overexpression significantly affected apoptosis or viability in SUDHL‐4 cells (Figure S3A–C). To evaluate miR‐19a‐3p's immunomodulatory effects, we established an indirect co‐culture system consisting of SUDHL‐4 cells and activated CD4^+^ T cells separated by transwell chambers (Figure 3A). Following co‐culture, the HBx‐overexpressing SUDHL‐4 cells exhibited a lower apoptosis rate than control cells, whereas miR‐19a‐3p overexpression reversed this HBx‐induced suppression (Figure 3B). Additionally, HBx‐engineered SUDHL‐4 cells triggered significant CD4^+^ T cell apoptosis, an effect that was effectively reversed by miR‐19a‐3p reconstitution (Figure 3C). The HBx‐mediated immunosuppression extended to lymphocyte replicative capacity and IFN‐γ/Granzyme B secretion, with both deficits rectified through miRNA overexpression (Figure 3D–F). These results identified miR‐19a‐3p as a non‐redundant orchestrator of CD4^+^ T cell‐mediated tumour surveillance in DLBCL.

### 3.4 MiR‐19a‐3p enhances CD4^+^ T cell‐mediated suppression of HBx‐expressing tumour in vivo

**FIGURE 4:**
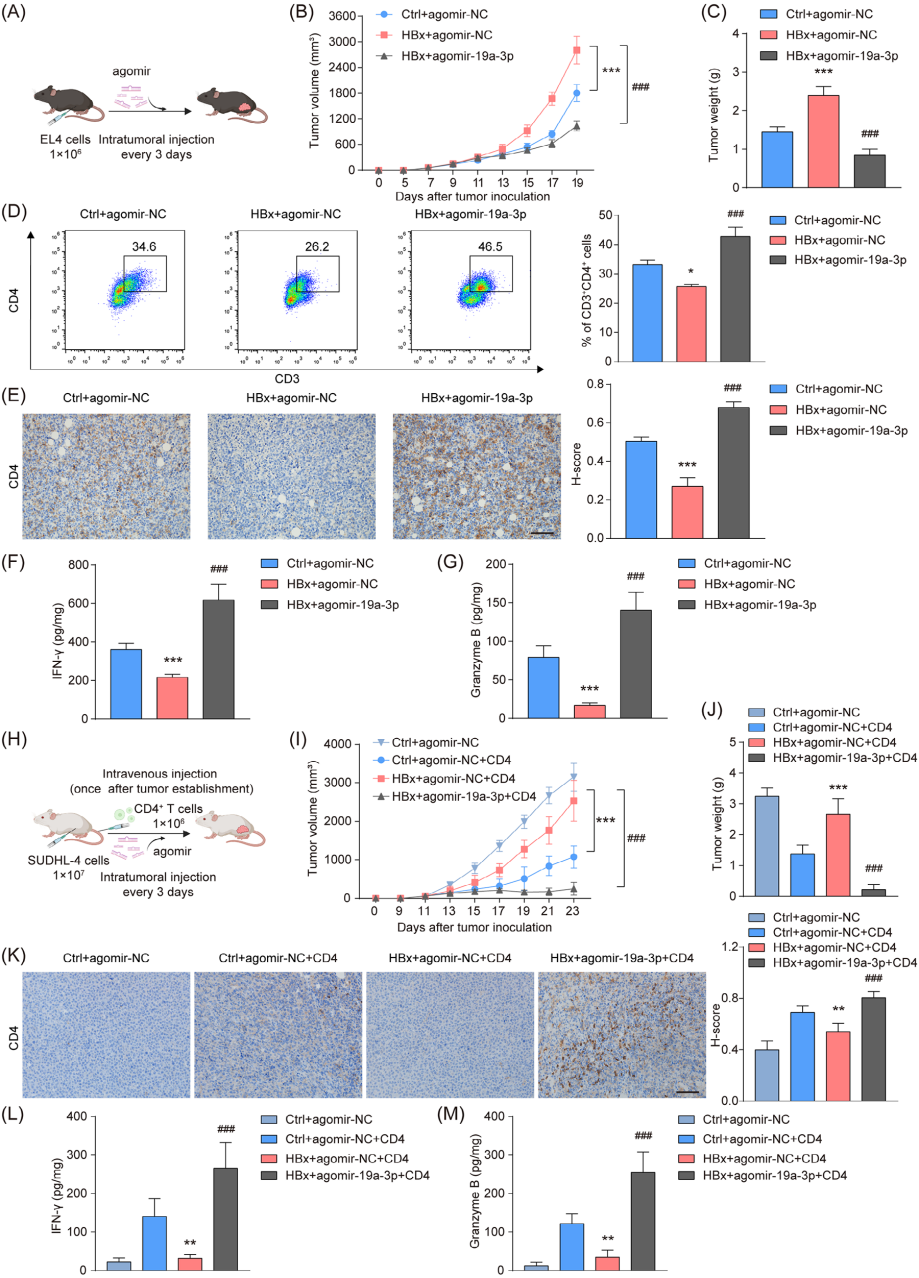
MiR‐19a‐3p enhances CD4^+^ T cell‐mediated suppression of HBx‐expressing tumour in vivo. (A) Schematic diagram of tumour cell transplantation and agomir injection. (B) MiR‐19a‐3p significantly abrogated the higher tumour growth rate caused by HBx. Tumour growth curves of the indicated groups were measured every 2 days (*n* = 6 for each group). (C) Tumour weights were measured at day 19. (D) Flow cytometry analysis to detect the subpopulations of CD4^+^ T cells in tumour tissues collected from a subcutaneous injection model of EL4 mice. In all experiments, cells were first gated on the CD45^+^ population and then on the CD4^+^ population. (E) The CD4^+^ T cell percentage was increased under miR‐19a‐3p treatment, as shown by IHC analysis. Scale bar = 100 µm. (F, G) The levels of IFN‐γ (F) and Granzyme B (G) in the tumour tissues were measured by ELISA. (H) Schematic diagram of humanized SUDHL‐4 xenograft model with adoptive transfer of human CD4⁺ T cells and agomir injection. (I) Tumour growth curves of indicated groups measured every 2 days (*n* = 6 for each group). (J) Tumour weights of indicated groups were measured at day 23. (K) IHC analysis of CD4⁺ T cell infiltration in SUDHL‐4 tumour tissues. Scale bar = 100 µm. (L, M) ELISA quantification of IFN‐γ (L) and Granzyme B (M) in SUDHL‐4 tumour tissues. The data represent the mean ± SD of six independent experiments. For panels B‐G, *HBx+agomir‐NC vs. Ctrl+agomir‐NC; #HBx+agomir‐19a‐3p vs. HBx+agomir‐NC. For panels I‐M, *HBx+agomir‐NC+CD4 vs. Ctrl+agomir‐NC+CD4; #HBx+agomir‐19a‐3p+CD4 vs. HBx+agomir‐NC+CD4. *p < .05, **p < .01, ***/^###^ *p* < .001.

Further, EL4 lymphoma cells were engrafted subcutaneously into syngeneic mice, followed by intratumoural administration of miR‐19a‐3p agomir or its control (Figure 4A). Compared with the HBx‐overexpressing group, miR‐19a‐3p suppressed tumour growth, as demonstrated by significant reductions in tumour volume and weight (Figure 4B,C). HBx overexpression markedly decreased tumour‐infiltrating CD4^+^ T cells, whereas miR‐19a‐3p agomir restored their infiltration (Figure 4D,E). Functionally, miR‐19a‐3p agomir rescued the HBx‐mediated suppression of IFN‐γ and Granzyme B expression (Figure 4F,G).

To further assess the impact of HBx and miR‐19a‐3p on human CD4^+^ T cell function in vivo, human DLBCL cells (SUDHL‐4) were engrafted into NOD/SCID mice, followed by the adoptive transfer of human CD4^+^ T cells (Figure 4H). Tumours established without CD4^+^ T cell transfer exhibited accelerated growth and higher tumour burden compared with those receiving CD4^+^ T cells (Figure 4I,J). Among mice reconstituted with CD4^+^ T cells, the HBx‐overexpressing tumours showed increased growth kinetics and final weights (Figure 4I,J), which were accompanied by reduced intratumoural CD4^+^ T cell infiltration and decreased IFN‐γ and Granzyme B levels (Figure 4K–M). Then, the miR‐19a‐3p agomir treatment restored CD4^+^ T cell infiltration and effector molecule production, along with reduced tumour growth in HBx‐overexpressing xenografts (Figure 4I–M). Collectively, these data demonstrated that miR‐19a‐3p potentiates CD4^+^ T cell‐mediated tumour surveillance and suppresses HBx‐driven oncogenic progression in both syngeneic and human xenograft lymphoma models.

### 3.5 BAMBI serves as the target gene of miR‐19a‐3p

**FIGURE 5:**
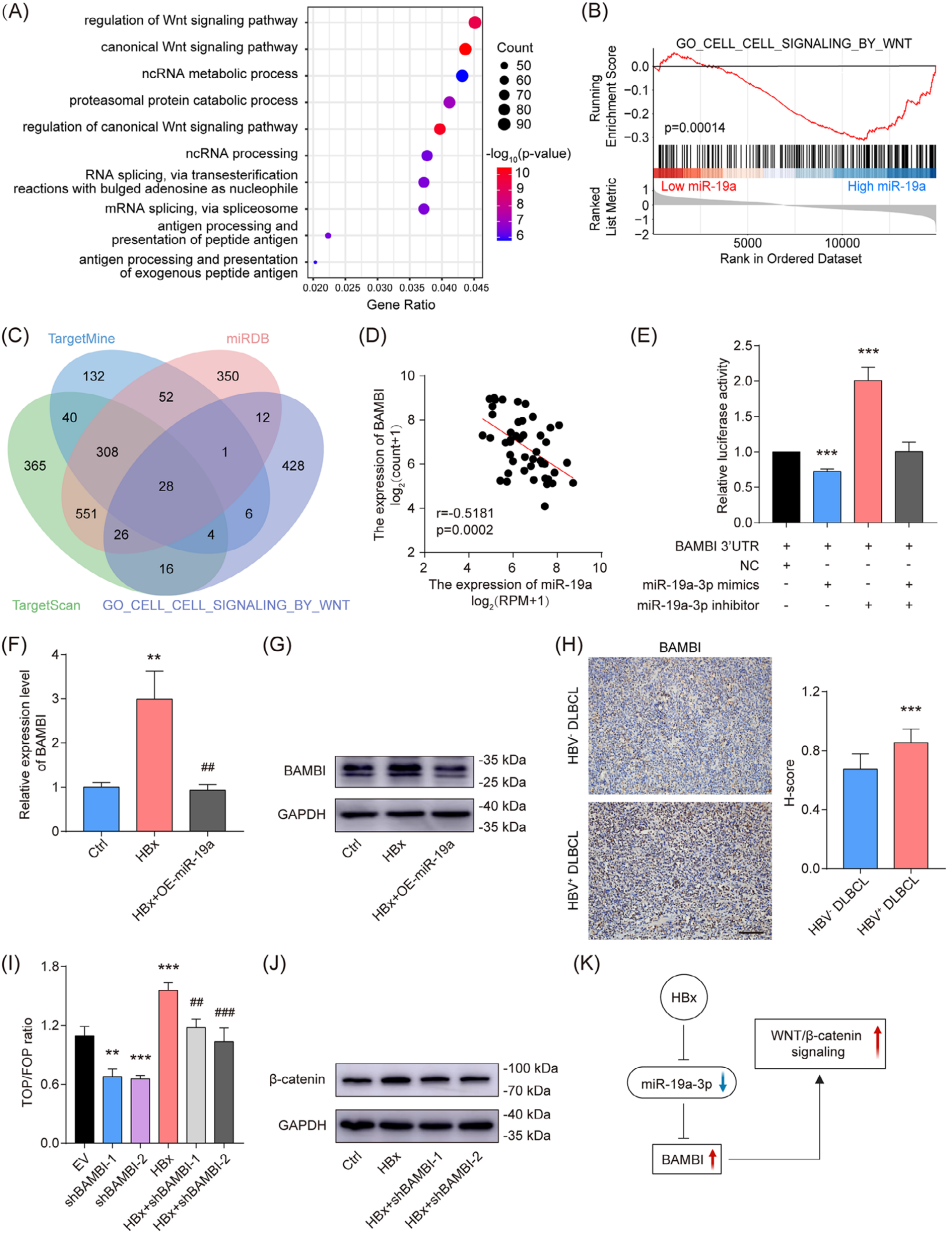
BAMBI serves as the target gene of miR‐19a‐3p. (A) GO enrichment analysis (TCGA‐DLBC). (B) Cell‐cell signalling by Wnt enrichment in GSEA (TCGA‐DLBC). (C) An intersection of the miR‐19a‐3p predicted targets from 3 prediction algorithms (TargetMine, miRDB, and TargetScan) and Wnt signalling‐related genes. (D) Correlation analysis between miR‐19a and BAMBI in DLBCL tissues (TCGA‐DLBC). (E) The effect of miR‐19a‐3p on transcriptional activity of BAMBI 3′UTR was detected by luciferase reporter assay. (F, G) The BAMBI expression regulated by HBx/miR‐19a was confirmed by qRT‐PCR (F) and western blot (G). (H) IHC analysis of BAMBI in the DLBCL tumour tissues. Scale bar = 100 µm. (I) Overall inductive effect of HBx and BAMBI on the Wnt/β‐catenin signalling was detected by TOP/FOP‐Flash reporter assay. (J) The expression of β‐catenin was detected by western blot in SUDHL‐4 cells. (K) Schematic model of the HBx/miR‐19a/BAMBI/Wnt axis. The data represent mean ± SD of three independent experiments. **/^##^ *p* < .01, ***/^###^ *p* < .001.

Putative targets of miR‐19a‐3p were computationally annotated through integrated Gene Ontology (GO) terms and GSEA of RNA‐seq data derived from the TCGA‐DLBC cohort. The top 10 GO terms, including regulation of the canonical Wnt signalling pathway, were shown (Figure 5A). GSEA revealed significant negative enrichment of canonical Wnt signalling gene signatures in subjects exhibiting elevated miR‐19a level (Figure 5B). By intersecting miR‐19a‐3p targets predicted by three algorithms (TargetMine, miRDB, and TargetScan) with genes in the ‘GO_CELL_CELL_SIGNALING_by_WNT’ pathway, we identified 28 high‐confidence candidates (Figure 5C). Survival analysis of patients stratified by these genes revealed 5 genes (BAMBI, GSKIP, CALM1, CAPRIN2, and PFN1) significantly associated with 3‐year survival, with BAMBI showing the strongest inverse correlation with miR‐19a levels (Figure 5D; Figure S4A,B). Dual‐luciferase reporter assays confirmed that miR‐19a‐3p mimics notably attenuated luminescence signals from BAMBI 3′UTR constructs, whereas miR‐19a‐3p inhibitors rescued this suppression (Figure 5E). Furthermore, miR‐19a overexpression reversed HBx‐induced BAMBI up‐regulation in SUDHL‐4 cells (Figure 5F,G). Compared with HBV^−^ DLBCL tissues, the expression of BAMBI was elevated in the HBV^+^ DLBCL tissues (Figure 5H). The results suggested that BAMBI is a downstream target gene of miR‐19a‐3p in DLBCL cells. TOP/FOP‐Flash assays confirmed that both HBx and BAMBI co‐regulated the activity of the Wnt/β‐catenin pathway (Figure 5I). Consistently, BAMBI knockdown markedly decreased β‐catenin levels in the HBx‐overexpressing SUDHL‐4 cells (Figure 5J). These results indicated that the HBx/miR‐19a‐3p axis modulates Wnt/β‐catenin signalling via targeting BAMBI (Figure 5K).

### 3.6 The miR‐19a‐3p/BAMBI axis regulates the anti‐tumour activity of CD4^+^ T cells

**FIGURE 6:**
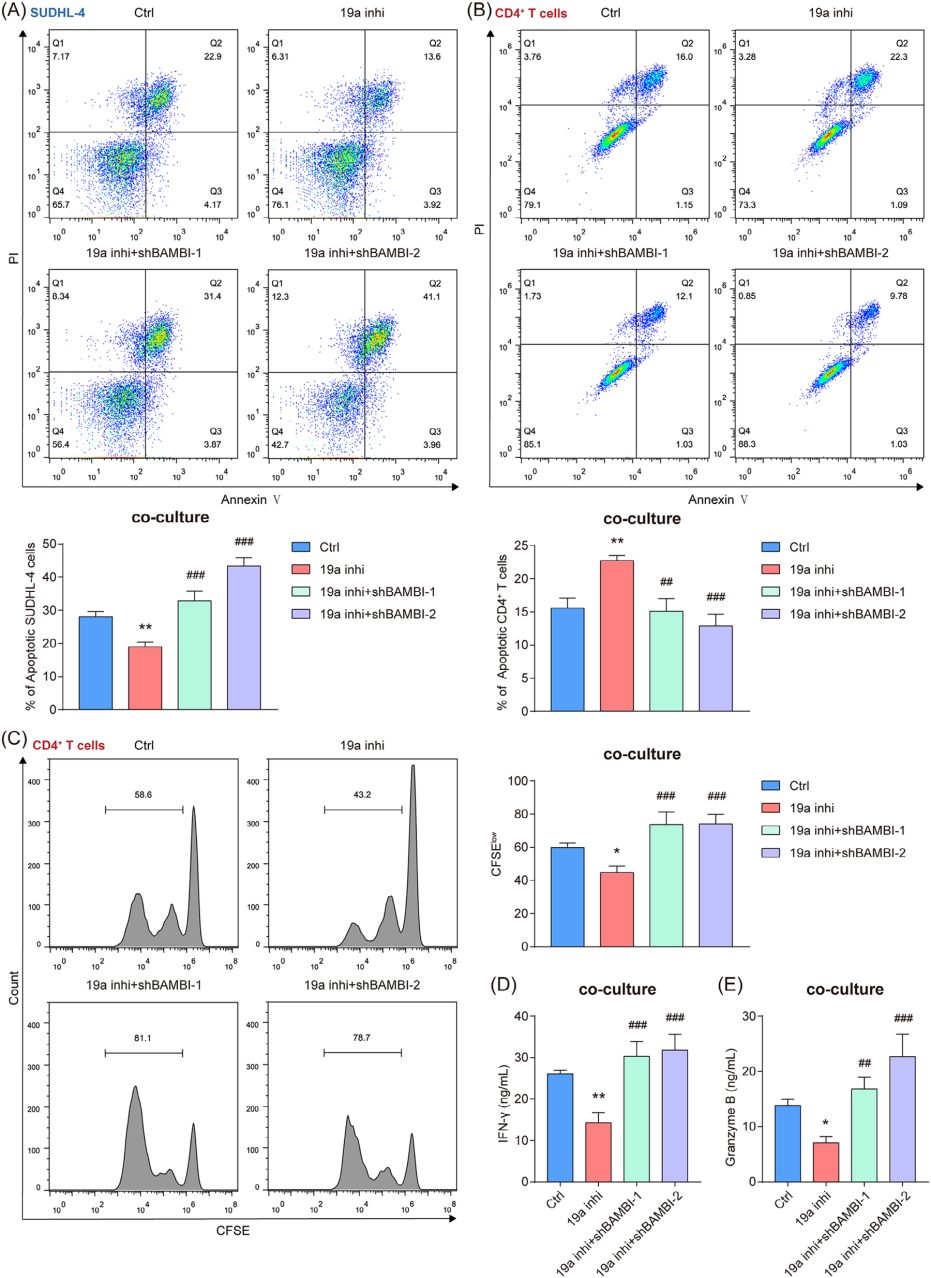
The miR‐19a‐3p/BAMBI axis regulates the anti‐tumour activity of CD4^+^ T cells. (A) Flow cytometry analysis for the changes in the proportion of apoptotic SUDHL‐4 cells within the co‐culture system. (B, C) Flow cytometry analysis for the changes in the proportions of apoptotic (B) and proliferative (C) CD4^+^ T cells in the co‐culture system, assessed by Annexin V‐FITC/PI staining and CFSE assay, respectively. (D, E) The levels of IFN‐γ (D) and Granzyme B (E) in the supernatant were measured by ELISA. The data represent mean ± SD of three independent experiments. *p < .05, **/^##^ *p* < .01, ^###^ *p* < .001. 19a inhi: miR‐19a‐3p inhibitor.

To determine the effect of miR‐19a‐3p/BAMBI on CD4^+^ T cells, we observed that miR‐19a‐3p antagonism reduced SUDHL‐4 apoptosis in co‐cultures, whereas BAMBI knockdown attenuated this anti‐apoptotic effect (Figure 6A). Notably, knockdown of BAMBI mitigated the pro‐apoptotic and anti‐proliferative effects on CD4^+^ T cells that were elicited by miR‐19a‐3p inhibition in SUDHL‐4 co‐cultures. (Figure 6B,C). This rescue phenotype extended to CD4^+^ T cell function: miR‐19a‐3p inhibitor‐treated SUDHL‐4 cells attenuated IFN‐γ and Granzyme B secretion from activated CD4^+^ T cells, effects counteracted by BAMBI knockdown (Figure 6D,E). Parallel experiments in HBx‐overexpressing SUDHL‐4 cells recapitulated these findings, with BAMBI knockdown restoring CD4^+^ T cell apoptosis, proliferation, and effector molecule secretion (Figure S5A–E). Critically, BAMBI knockdown did not intrinsically alter HBx‐SUDHL‐4 cell apoptosis or viability (Figure S6A,B), confirming that the observed immune modulation was T cell‐dependent. Together, these data established BAMBI as the mechanistic bridge through which miR‐19a‐3p regulates DLBCL‐CD4^+^ T cell interactions.

### 3.7 The DLBCL‐derived TGF‐β1 translates HBx/miR‐19a‐3p/BAMBI signalling into CD4^+^ T cell exclusion and immune escape

**FIGURE 7:**
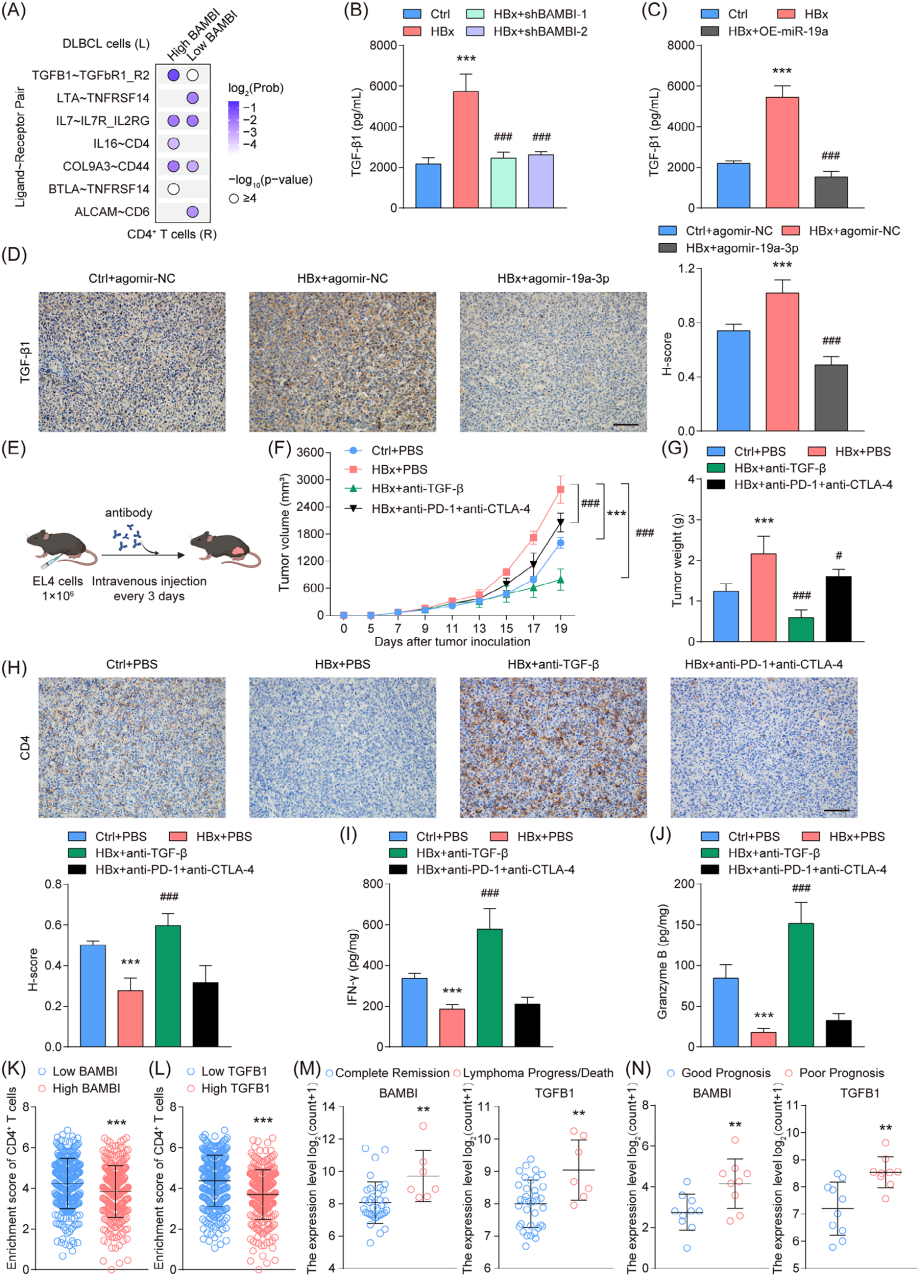
The DLBCL‐derived TGF‐β1 translates HBx/miR‐19a‐3p/BAMBI signalling into CD4^+^ T cell exclusion and immune escape. (A) Ligand (L) and receptor (R) counts in significant DLBCL‐CD4^+^ T cell interactions (GSE182434). (B, C) The expression level of TGF‐β1 were measured by ELISA. (D) IHC analysis of TGF‐β1 in the EL4 tumour tissues. Scale bar = 100 µm. (E) Schematic diagram of tumour cell transplantation and antibody injection. (F) Tumour growth curves of EL4 tumours in mice. (G) Tumour weights were measured at day 19. (H) CD4^+^ T cell infiltration in tumour tissues determined by IHC. Scale bar = 100 µm. (I, J) The levels of IFN‐γ (I) and Granzyme B (J) in the tumour tissues were measured by ELISA. (K, L) High expression level of BAMBI (K) or TGFB1 (L) correlated with reduced CD4^+^ T cells enrichment (GSE125966). (M) Patients with poor response to R‐CHOP treatment exhibited higher expression of BAMBI and TGFB1 (GSE23501). (N) Compared with patients with favourable outcomes (durable remission ≥4 years), those with poor prognosis (primary refractory or relapsed disease) showed significantly higher expression of BAMBI and TGFB1 (GSE178965). The data represent mean ± SD of at least three independent experiments. **p < .01, ***/^###^ *p* < .001.

To systematically characterize the tumour cell‐CD4^+^ T cell communication network, we performed ligand–receptor (L‐R) pair analysis leveraging single‐cell transcriptomic profiles from 4 DLBCL cases. Through integrated analysis of 3473 B‐cell copy number variation (CNV) profiles and canonical DLBCL markers (Figure S7A,B), we classified these cells into 2,905 malignant B cells and 568 tumour‐infiltrating B lymphocytes within the TME. Subsequent stratification of malignant B cells by BAMBI expression revealed seven potential L‐R pairs governing DLBCL‐CD4^+^ T cell interactions (Figure 7A). Consistent with the predicted ligand–receptor signalling, TGFBR2 knockdown in CD4^+^ T cells increased HBx‐overexpressing lymphoma cell apoptosis, while reducing CD4^+^ T cell apoptosis and enhancing proliferation and effector cytokine secretion (Figure S8A–E). Mechanistically, HBx‐overexpressing SUDHL‐4 cells exhibited marked TGF‐β1 up‐regulation, which was reversed by either BAMBI knockdown or miR‐19a‐3p overexpression (Figure 7B,C; Figure S9A,B). Notably, miR‐19a‐3p agomir treatment effectively abrogated HBx‐driven BAMBI/TGF‐β1 activation (Figure 7D; Figure S10A,B), demonstrating the miR‐19a‐3p/BAMBI axis as a key upstream regulator of TGF‐β1. These findings positioned the TGFB1‐TGFBR2 pair as a central mediator of immune crosstalk between malignant B cells and CD4^+^ T lymphocytes in DLBCL pathogenesis.

To further validate the predicted regulatory relationships and directly test the functional role of the miR‐19a‐3p/BAMBI/TGF‐β1 axis, the HBx‐overexpressing SUDHL‐4 cells were subjected to CRISPR/Cas13d‐mediated knockdown of miR‐19a‐3p and CRISPR/Cas9‐mediated knockout of BAMBI or TGFB1. Our findings showed that knockdown of miR‐19a‐3p led to a more significant impact on the induction of BAMBI and TGFB1 expression and suppression of CD4^+^ T cell activity, whereas knockout of BAMBI or TGFB1 significantly reversed these effects, confirming their roles as key downstream mediators (Figure S11A–H). These results provided direct functional and mechanistic evidence that HBx regulates CD4^+^ T cell activity via the miR‐19a‐3p/BAMBI/TGFB1 pathway.

To assess the therapeutic efficacy of TGF‐β blockade in comparison with immune checkpoint inhibition, EL4 lymphoma‐bearing mice were treated with anti‐TGF‐β antibody or a combination of anti‐PD‐1 and anti‐CTLA‐4 antibodies (Figure 7E). TGF‐β blockade markedly suppressed the growth of HBx‐overexpressing tumours, whereas PD‐1/CTLA‐4 dual blockade exerted only modest effects (Figure 7F,G). Treatment with TGF‐β antibody restored intratumoural CD4^+^ T cell infiltration without affecting BAMBI or TGF‐β1 expression (Figure 7H; Figure S12A,B). Moreover, the impaired secretion of cytotoxic molecules (IFN‐γ and Granzyme B) by activated CD4^+^ T cells was reversed by TGF‐β antibody treatment in HBx‐overexpressing tumours (Figure 7I,J). By contrast, PD‐1/CTLA‐4 dual blockade neither restored CD4^+^ T cell infiltration nor enhanced IFN‐γ and Granzyme B production (Figure 7H–J), consistent with its limited anti‐tumour activity. Collectively, these results mechanistically linked the miR‐19a‐3p/BAMBI/TGF‐β1 axis to CD4^+^ T cell dysfunction in HBx‐overexpressing DLBCL and provided preclinical evidence for TGF‐β‐targeted immunotherapy to counteract immune escape.

To evaluate the clinical implications of BAMBI and TGFB1, we analyzed integrated multi‐cohort datasets. In the GOYA cohort, high expression of BAMBI correlated with diminished CD4^+^ T cell enrichment in tumour tissues, a trend mirrored by TGFB1 (Figure 7K,L). Clinically, progressive or deceased DLBCL patients exhibited markedly higher levels of BAMBI and TGFB1 in tumour tissues compared with complete responders (Figure 7M,N). OncoPredict‐based drug sensitivity modelling further revealed reduced chemotherapeutic responsiveness in the BAMBI/TGFB1 high‐expression group (Figure S13A,B). Collectively, these results suggested that BAMBI/TGFB1 may modulate DLBCL clinical outcomes by regulating immune escape and chemotherapy resistance.

### 3.8 BAMBI^high^ DLBCL remodels CD4^+^ T cell phenotypes via TGFB1‐TGFBR2 pair

**FIGURE 8:**
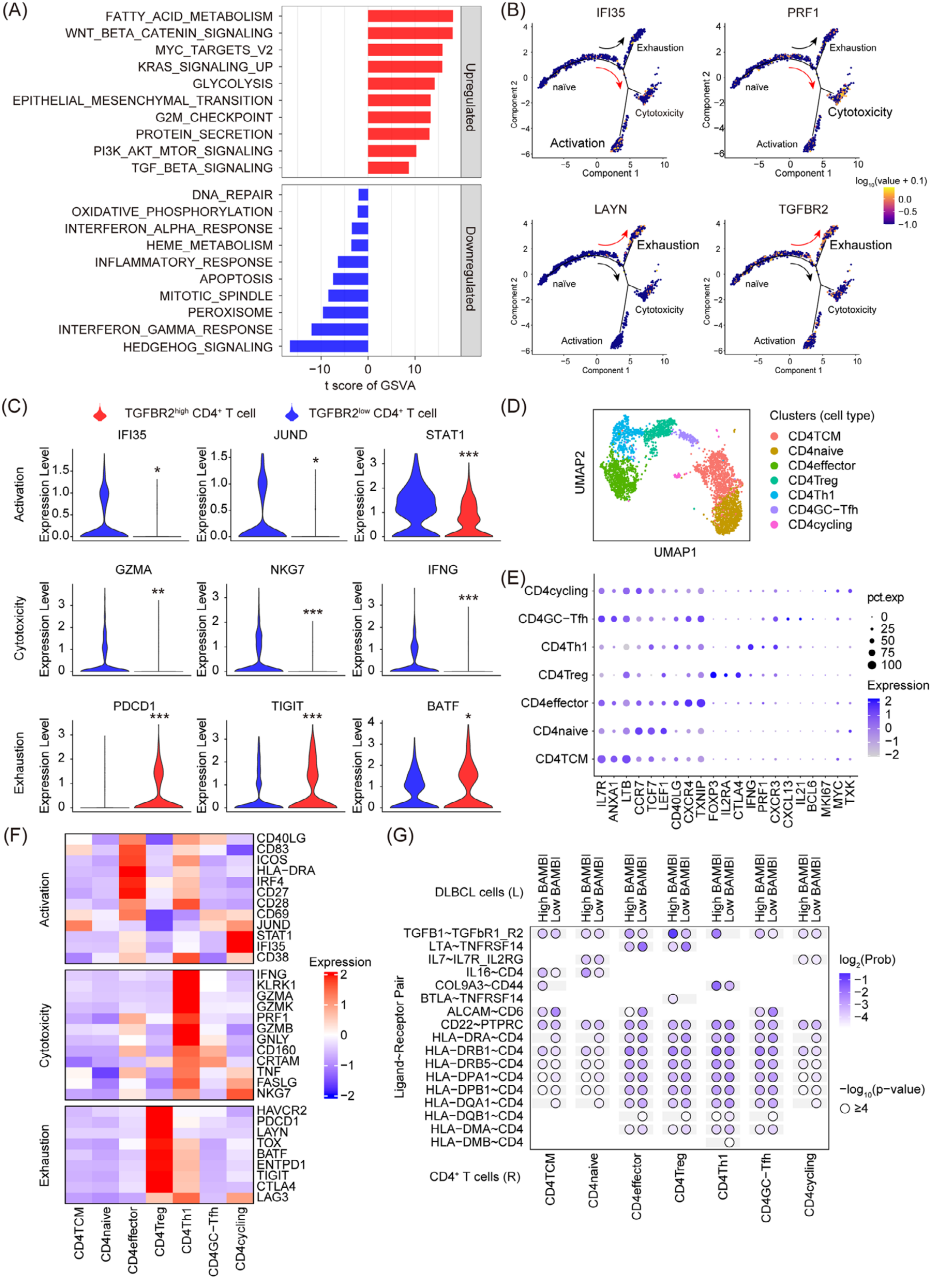
BAMBI^high^ DLBCL remodels CD4^+^ T cell phenotypes via TGFB1‐TGFBR2. (A) GSVA enrichment analysis of hallmark pathways in BAMBI^high^TGFB1^high^ DLBCL cells (GSE182434). (B) Pseudotime trajectory of CD4^+^ T cell differentiation states (GSE182434). (C) Violin plot showing the expression profiles of indicated genes. (D) UMAP projection of the 3547 CD4^+^ T cells from 4 DLBCL patients. Each colour represents a CD4^+^ T cell cluster. (E) Expression levels of marker genes identified for the 7 CD4^+^ T cell clusters. (F) Functional profiles of the 7 CD4^+^ T cell clusters. (G) The number of regulatory axes between DLBCL and CD4^+^ T cell clusters. *p < .05, **/^##^ *p* < .01, ***/^###^ *p* < .001.

To validate transcriptomic findings, we further performed scRNA‐seq analysis of malignant B cells and CD4^+^ T cells, identifying BAMBI‐TGFB1 co‐expression in malignant B cells (Figure S14A,B). The GSVA analysis revealed activation of Wnt signalling, protein secretion, and the TGF‐β pathway, but suppression of IFN‐γ response, inflammation, and apoptosis in BAMBI^high^TGFB1^high^ DLBCL cells (Figure 8A). Considering that BAMBI^high^TGFB1^high^ DLBCL cells actively secrete TGF‐β1, we investigated how its sensing by CD4^+^ T cells through TGFBR2 contributes to their fate determination in the TME. To this end, we constructed a differentiation trajectory for CD4^+^ T cells, tracing their transition from naïve to activated, cytotoxic, or exhausted phenotypes, and found that TGFBR2 expression was mainly confined to the exhausted lineage (Figure 8B). TGFBR2^high^ CD4^+^ T cells exhibited reduced activation (IFI35, JUND, and STAT1) and cytotoxicity (GZMA, NKG7, and IFNG) markers, but enhanced exhaustion (PDCD1, TIGIT, and BATF; Figure 8C). These results established TGFBR2 as a central regulator of CD4^+^ T cell exhaustion programming in DLBCL microenvironments.

To investigate the CD4^+^ T cell subtypes primarily regulated by DLBCL cells through the TGFB1‐TGFBR2 pair, we identified and characterized 7 clusters from 3,547 intratumoural CD4^+^ T cells based on marker gene expression (Figure 8D,E). The CD4_effector_ cluster showed strong activation characteristics, the CD4_Treg_ cluster showed a strong exhaustion phenotype, while the CD4_Th1_ cluster showed strong cytotoxic properties (Figure 8F). Ligand–receptor interaction analysis showed that compared with BAMBI^low^ DLBCL cells, BAMBI^high^ DLBCL cells had stronger TGFB1‐TGFBR2 pair interactions with CD4_effector_, CD4_Treg,_ and CD4_Th1_ clusters (Figure 8G). In summary, our results demonstrated that BAMBI^high^ DLBCL cells may tend to suppress the anti‐tumour activity of CD4 effector, regulatory, and Th1 subsets by enhancing TGFB1‐TGFBR2 signalling in the TME.

## 4 DISCUSSION

Emerging evidence underscores that the immune composition of the TME is a key determinant of DLBCL prognosis. Previous studies in DLBCL patients have shown that peritumoural dendritic cell accumulation and intratumoural CD8^+^ T cell infiltration are associated with favourable outcomes,^33^ while higher intratumoural CD4^+^ T cell density correlates with improved post‐chemotherapy survival.^34^, ^35^ These findings highlight the essential roles of both cytotoxic and helper T cell subsets in shaping therapeutic responses. In HBV^+^ DLBCL, systemic immune alterations provide further support for this paradigm: a lower absolute lymphocyte count (ALC) in peripheral blood predicts poor chemotherapy response,^36^, ^37^, ^38^ and peripheral CD4^+^ T cell levels decline markedly during chemotherapy in HBV‐related DLBCL patients.^39^ Our study extended these observations by providing, for the first time, direct evidence that HBV infection reduces intratumoural CD4^+^ T cell abundance, which is closely linked to inferior survival and adverse clinical outcomes. Beyond this quantitative loss, our data further revealed that HBV compromises the anti‐tumour capacity of CD4^+^ T cells, thereby imposing an additional layer of immune suppression that aggravates immune evasion and accelerates disease progression. Collectively, these findings established CD4^+^ T cell loss as a central driver of poor prognosis in HBV^+^ DLBCL and pointed to the restoration of CD4^+^ T cell‐mediated immunity as a rational therapeutic strategy with potential clinical impact.

Several studies have investigated HBV‐regulated microRNAs in HCC, revealing that dysregulation of miR‐152, miR‐106b, and miR‐19a contributes to immune suppression and T cell exhaustion,^40^, ^41^, ^42^, ^43^ establishing a mechanistic connection between viral regulation and anti‐tumour immunity.^44^ Building on these insights, our study extended the concept to lymphoma, identifying miR‐19a‐3p as a novel HBx‐regulated miRNA that directly modulated CD4^+^ T cell function in DLBCL. Functional assays confirmed that miR‐19a‐3p enhances CD4^+^ T cell cytotoxicity and counteracts HBx‐driven lymphomagenesis, establishing a mechanistic connection between viral regulation and anti‐tumour immunity. These findings align with previous studies suggesting a broader role for HBV‐mediated miRNA networks in shaping adaptive immunity.^45^, ^46^ Importantly, miR‐19a‐3p may also represent a novel diagnostic and prognostic molecular marker to aid in the identification and stratification of HBV^+^ DLBCL, complementing conventional virological assays (HBsAg, HBV DNA) and immunohistochemical detection of viral proteins.^47^, ^48^ However, we employed an HBx‐overexpressing DLBCL model rather than a full HBV infection system. Since NTCP is restricted to hepatocytes,^49^ the entry receptors of HBV in DLBCL remain unknown, warranting future studies to clarify alternative pathways.

Intercellular signalling is pivotal for HBV‐driven TME remodelling, a process mediated by pathway hijacking.^50^ Here, we identified BAMBI as a direct miR‐19a‐3p target, establishing a functional link between HBV infection and Wnt/β‐catenin signalling in DLBCL. Notably, Wnt/β‐catenin activation did not enhance lymphoma cell proliferation but instead induced up‐regulation of TGF‐β1, a well‐recognized mediator of immunosuppression.^51^ This observation was consistent with prior reports indicating that TGF‐β impedes T cell proliferation, regulates effector activity, and limits immune surveillance.^52^, ^53^, ^54^ Single‐cell transcriptomic profiling further revealed that TGFBR2 expression was enriched in exhausted CD4^+^ T cell subsets, supporting its role in mediating T cell dysfunction in HBV^+^ DLBCL. Consistently, TGFBR2 knockdown restored CD4^+^ T cell anti‐tumour activity against DLBCL cells. Moreover, DLBCL cells with elevated BAMBI expression exhibited intensified interactions with effector, regulatory, and Th1 CD4^+^ T cell subsets via the TGFB1‐TGFBR2 ligand–receptor axis, delineating a specific intercellular mechanism of immunosuppression. Collectively, these findings identified the BAMBI‐Wnt‐TGF‐β axis as a novel pathway by which HBV results in the CD4^+^ T cell exhaustion in lymphoma. Clinically, the high TGFB1 (tumour) and TGFBR2 (CD4^+^ T cells) expression were jointly associated with inferior survival in HBV^+^ DLBCL, highlighting their potential as complementary prognostic markers capturing tumour‐immune interactions, distincted from established clinical predictors such as IPI, cell‐of‐origin, and double‐hit status.^55^, ^56^ From a translational perspective, the relevance of this pathway is underscored by preclinical and early clinical studies in solid tumours demonstrating that TGF‐β blockade restores anti‐tumour immunity,^57^, ^58^, ^59^ suggesting potential applicability in HBV^+^ DLBCL. While our integrated approach strengthens these conclusions, the limited cohort size with transcriptomic data necessitates validation in larger prospective studies.

Prior studies have shown that PD‐1 and PD‐L1 expression are generally low in DLBCL,^60^ with PD‐L1 preferentially enriched in non‐GCB rather than GCB subtypes.^61^ This suggests that immune checkpoint inhibitors may lack sufficient ligand engagement in GCB‐DLBCL, thereby limiting therapeutic benefit. Clinical trials have reported modest responses to PD‐1 blockade in relapsed or refractory DLBCL,^62^, ^63^, ^64^ particularly in the GCB subtype.^65^ Similarly, CTLA‐4 inhibition—either alone or in combination with PD‐1 blockade—has yielded limited clinical activity in B‐cell lymphomas,^66^, ^67^, ^68^ further underscoring the restricted efficacy of checkpoint inhibition in this disease. Notably, we demonstrated for the first time that TGF‐β was highly expressed in both GCB and non‐GCB subtypes of DLBCL, and that it functioned as a dominant immunosuppressive axis specifically in the GCB context, consistented with our experimental findings. This mechanistic insight directly explained why TGF‐β blockade achieved superior anti‐tumour efficacy compared with PD‐1/CTLA‐4 inhibition in our in vivo model, highlighting the translational potential of targeting this pathway in GCB‐DLBCL. Nonetheless, clinical translation of these insights remains challenging. Future studies should leverage patient‐derived xenograft (PDX) models to assess the safety and efficacy of TGF‐β‐targeted therapies—such as NIS793, SAR439459, and PF‐06952229, currently under investigation in solid tumours^69^, ^70^, ^71^ —with promising preclinical results potentially guiding their clinical repurposing for PD‐1‐resistant HBV^+^ DLBCL. Additionally, efforts should focus on both molecular targeting approaches—including monoclonal antibodies and small‐molecule inhibitors, as well as complementary strategies such as miRNA agomirs—and delivery strategies (e.g., nanomaterial‐based systems) to achieve precise modulation of the miR‐19a‐3p/BAMBI/TGF‐β1 axis in the HBV^+^ DLBCL.

## 5 CONCLUSION

In summary, this study revealed that HBV infection suppresses miR‐19a‐3p in DLBCL, subsequently activating the Wnt‐TGF‐β signalling and compromising CD4^+^ T cell‐mediated anti‐tumour immunity through ligand–receptor crosstalk. Our work uncovers the previously undefined contribution of CD4^+^ T cell dysfunction to the aggressiveness of HBV^+^ DLBCL, providing a mechanistic basis for its poor prognosis. Given the therapeutic tractability of miRNAs, these findings highlight miR‐19a‐3p restoration as a strategy to disrupt immunosuppressive signalling and improve clinical outcomes in HBV‐associated DLBCL.

## AUTHOR CONTRIBUTIONS

*Conceptualization*: Jiuhong Kang, Jie Chen, and Xudong Guo. *Formal analysis and investigation*: Xuecong Guo, Jianguo Li, Xiaofei Bai, Yinghui Huang, Xu Xu, Jiabang Yang, Zhenghao Sun, and Wangcheng Zhu. *Writing original draft*: Xuecong Guo. *Writing review and editing and funding acquisition*: Jiuhong Kang and Xudong Guo. All authors contributed to the interpretation of the results and provided valuable feedback.

## CONFLICT OF INTEREST STATEMENT

The authors declare no conflict of interest.

## ETHICS STATEMENT

All procedures involving experimental animals were conducted in strict accordance with the guidelines and regulations of the Experimental Animal Center of Tongji University. All animal experiments were performed under protocols approved by the Animal Protection and Use Committee of Tongji University (Approval Number: TJAB04524105). The paraffin‐embedded cancer patient samples used in this study were obtained from Changhai Hospital of Naval Medical University. Informed consent was obtained from all participants, and the study was approved by the Ethics Committee of Changhai Hospital of Naval Medical University. Commercially obtained human PBMCs were used in this study; informed consent and ethical approval were obtained by the supplier.

## Supporting information



Supporting Information

Supporting Information

Supporting Information

## Data Availability

The authors declare that all relevant data of this study are available within the article or from the corresponding author upon request. The dataset supporting the conclusions of this article is included within the supplementary information file.
