# Supplementary material for: HBx hijacks the miR‐19a‐3p/BAMBI/TGF‐β1 axis to impair the anti‐tumour activity of CD4+ T cells in diffuse large B‐cell lymphoma
Source: Clin Transl Med. 2026 Jan 5;16(1):e70578. doi: 10.1002/ctm2.70578 (PMC12771663; doi:10.1002/ctm2.70578)
Supplement: Supplementary file 1 — Supporting Information [file CTM2-16-e70578-s002.docx]

**Supplementary Figures and Legends for**

HBx hijacks the miR-19a-3p/BAMBI/TGF-β1 axis to impair the anti-tumor activity of CD4^+^ T cells in diffuse large B-cell lymphoma

Xuecong Guo ^1^, Jianguo Li ^1^, Xiaofei Bai ^1^, Yinghui Huang ^1^, Xu Xu ^2^, Jiabang Yang ^2^, Zhenghao Sun ^1^, Wangcheng Zhu ^3^, Xudong Guo ^1*^, Jie Chen ^2*^ & Jiuhong Kang ^1*^

^1^Clinical and Translational Research Center of Shanghai First Maternity and Infant Hospital, Shanghai Key Laboratory of Maternal Fetal Medicine, Shanghai Key Laboratory of Signaling and Disease Research, Frontier Science Center for Stem Cell Research, National Stem Cell Translational Resource Center, School of Life Sciences and Technology, Tongji University, Shanghai, 200092, China. ^2^Department of Hematology, Changhai Hospital, Naval Medical University, Shanghai, 200433, China. ^3^Department of Life Sciences, Imperial College London, London, SW7 2AZ, UK

**Corresponding Author:** Jiuhong Kang (jhkang@tongji.edu.cn); Jie Chen (chenjiedoctor@163.com); Xudong Guo (19504@tongji.edu.cn)

**
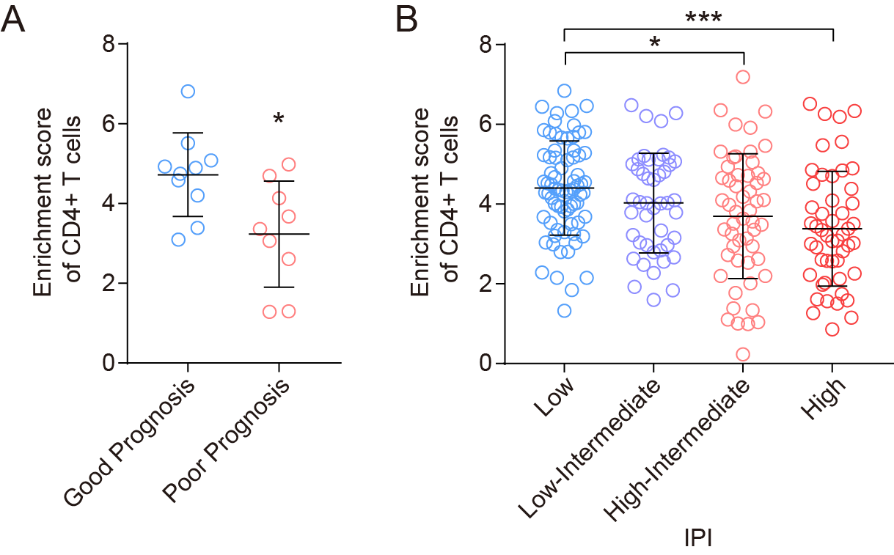
**

**Supplementary Figure 1. The CD4^+^ T cell enrichment in the TME of DLBCL predicts clinical outcomes.**

(A) The CD4^+^ T cell enrichment scores in DLBCL patients grouped by clinical outcome (good prognosis *vs*. poor prognosis, GSE178965). (B) The CD4^+^ T cell enrichment scores in DLBCL tissues stratified by IPI risk groups (GSE87371). The error bars represented the mean ± SD. *p < 0.05, ***/###p < 0.001.

**
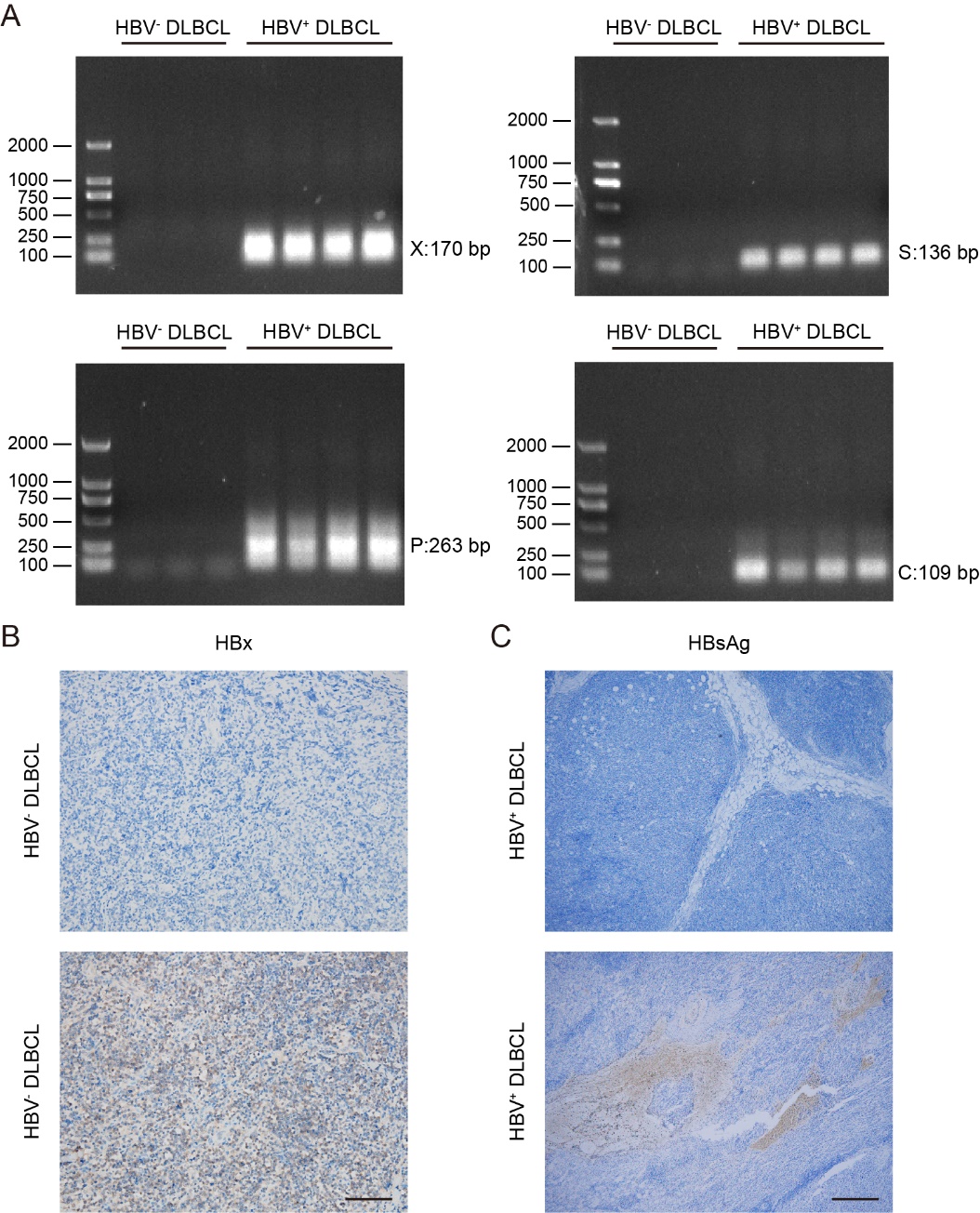
**

**Supplementary Figure 2**. **The detection of HBV genomic fragments and HBx viral proteins in DLBCL tissues.**

(A) PCR amplification of four HBV genes (X, S, P, and C) in the HBV⁻ and HBV⁺ DLBCL tissues. (B) Representative immunohistochemical staining showing HBx protein expression in the DLBCL tissues. Scale bar = 100 μm. (C) Representative immunohistochemical staining showing HBsAg expression in the DLBCL tissues. Scale bar = 500 μm.

**
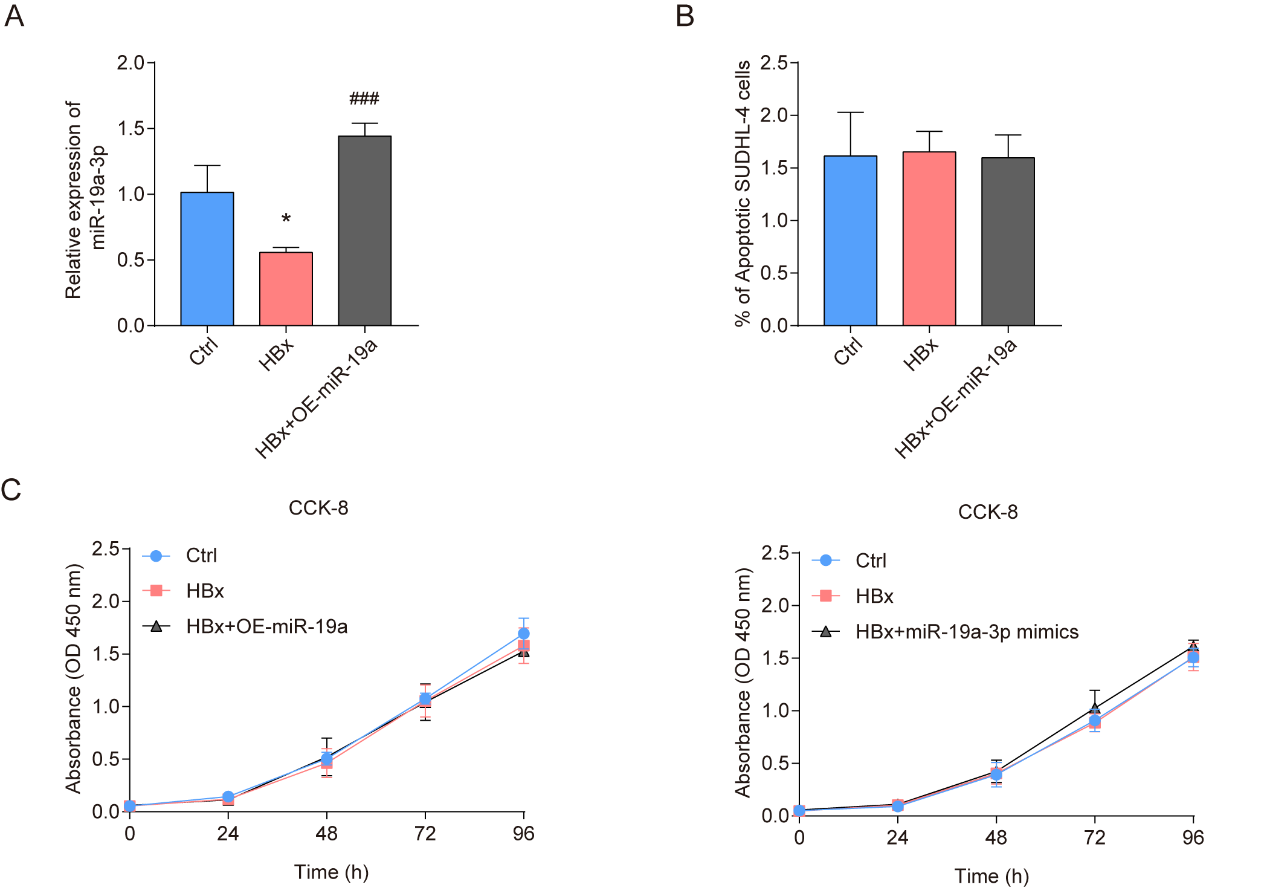
**

**Supplementary Figure 3**. **HBx and miR-19a-3p** **do not affect the proportions of proliferative and apoptotic DLBCL cells.**

(A) The expression of miR-19a-3p in DLBCL cells was determined by qRT-PCR. (B) Flow cytometry analysis of cell apoptosis using Annexin V-FITC/PI staining. (C) The proliferation capacity was measured by CCK8 assay in DLBCL cells. The data represent mean ± SD of three independent experiments. **p* < 0.05, and ###*p* < 0.001.


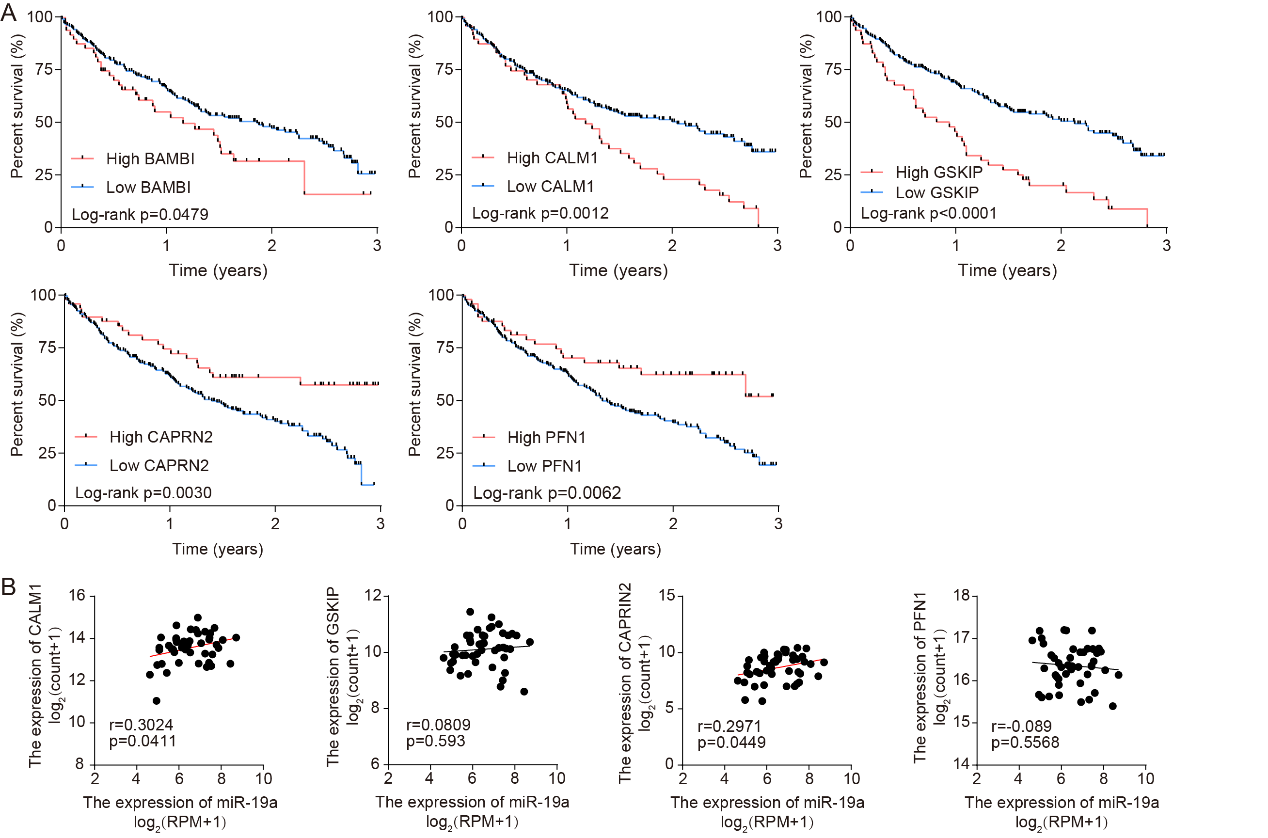


**Supplementary Figure 4. Screening for miR-19a-3p candidate target genes.**

(A) Survival analysis of the predicted target genes of miR-19a-3p in DLBCL patients (GSE10846). (B) Correlation analysis between miR-19a and its potential target genes in DLBCL tissues (TCGA-DLBC).


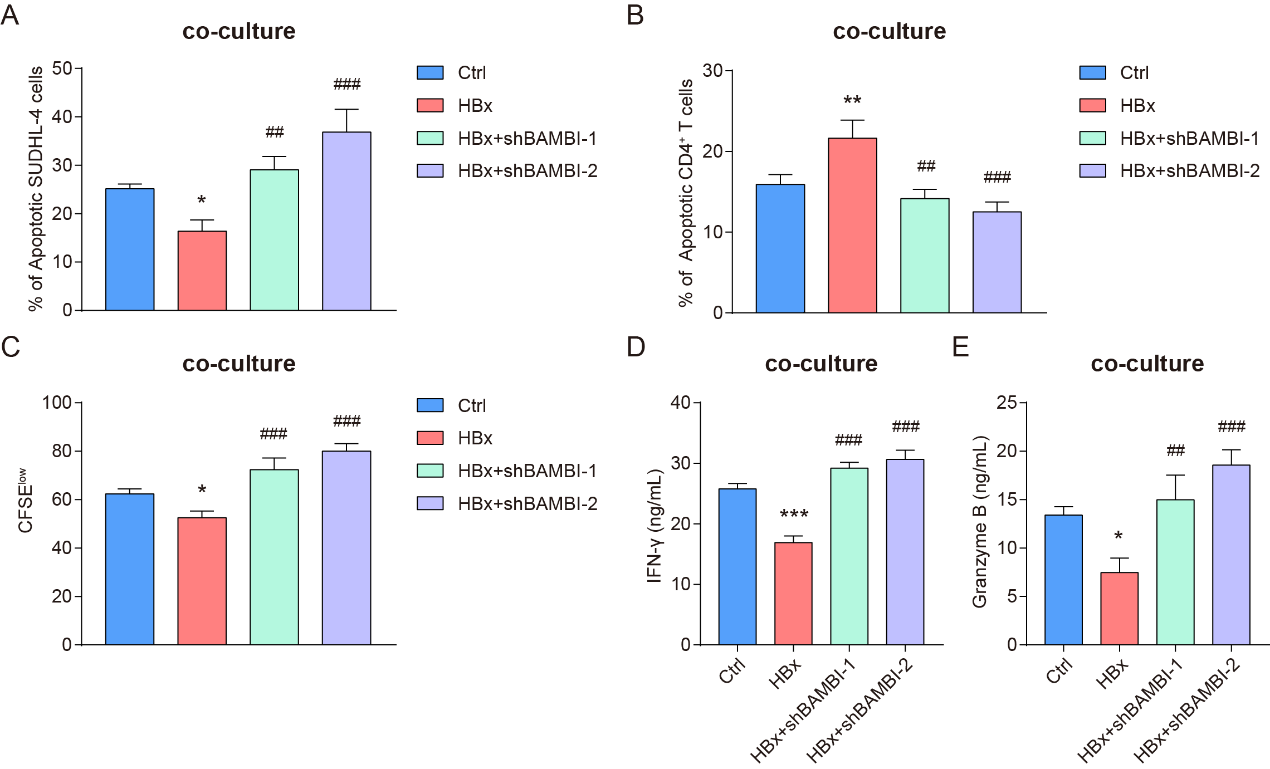


**Supplementary Figure 5. Knockdown of BAMBI increases the anti-tumor activity of CD4^+^ T cells against HBx-expressing DLBCLs.**

(A) Flow cytometry analysis for the changes in the proportion of apoptotic SUDHL-4 cells within co-culture system. (B, C) Flow cytometry analysis for the changes in the proportion of apoptotic (B) and proliferative (C) CD4^+^ T cells in co-culture system, assessed by Annexin V-FITC/PI staining and CFSE assay, respectively. (D, E) The IFN-γ (D) and Granzyme B (E) levels in the supernatant were measured by ELISA. The data represent mean ± SD of three independent experiments. **p* < 0.05, **/##*p* < 0.01, and ***/###*p* < 0.001.


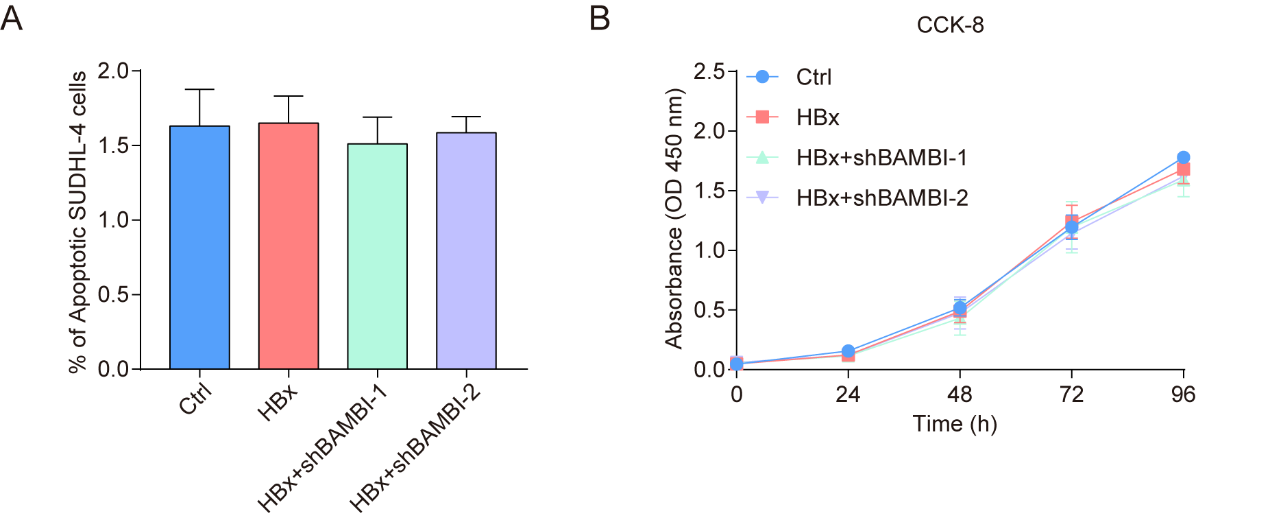


**Supplementary Figure 6. Knockdown of BAMBI does not affect the proliferative and apoptotic proportions of HBx-overexpressing DLBCL cells.**

(A) Flow cytometry analysis of cell apoptosis using Annexin V-FITC/PI staining in the HBx-overexpressing SUDHL-4 cells, with or without BAMBI knockdown. (B) The proliferation capacity was measured by CCK8 assay in the HBx-expressing SUDHL-4 cells, with or without BAMBI knockdown. The data represent mean ± SD of three independent experiments.


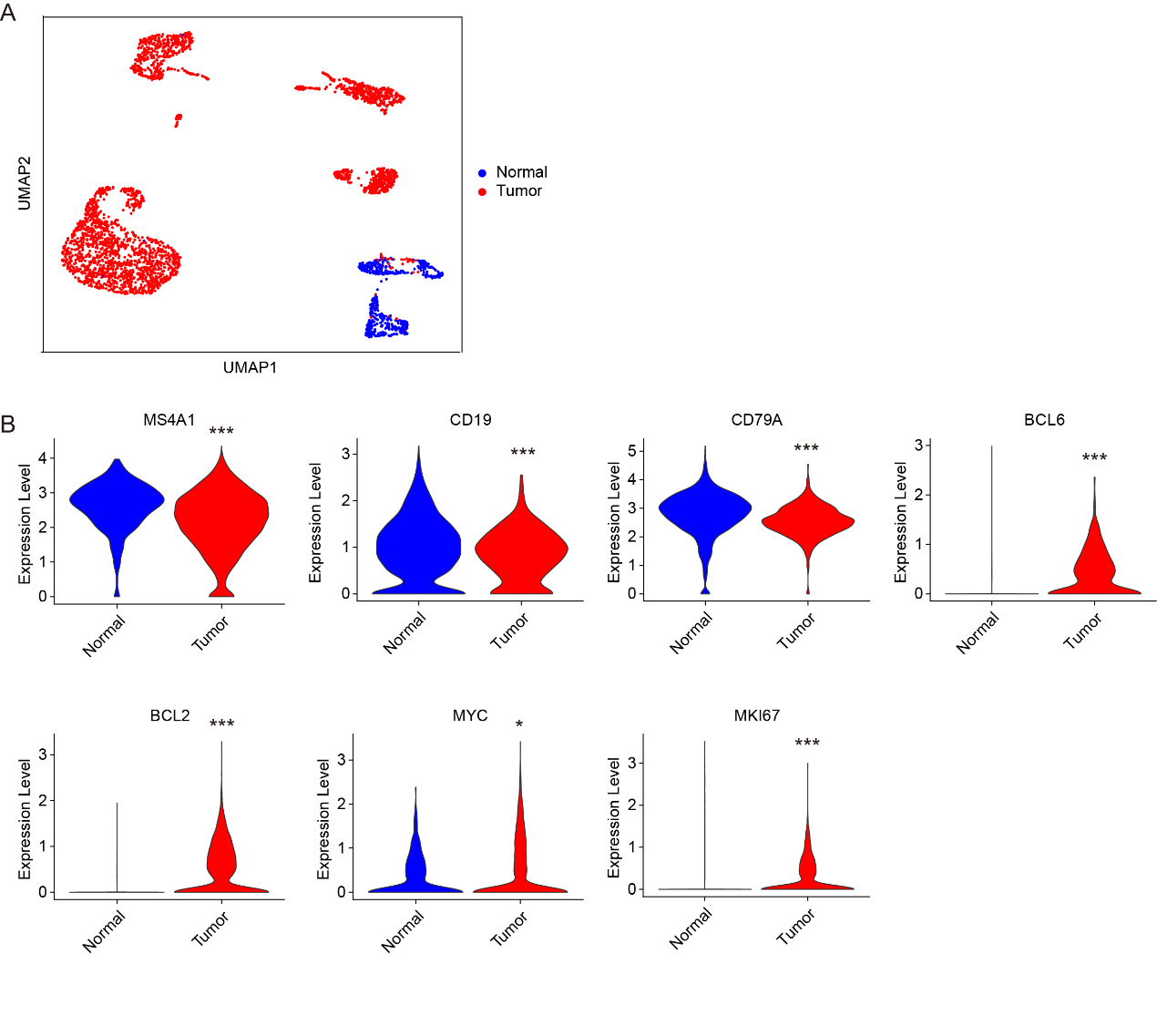


**Supplementary Figure 7. Genomic and molecular characterization of tumor versus normal infiltrating B cells in the TME of DLBCL.**

(A) UMAP of B cells (tumor B cells in red, normal infiltrating B lymphocytes in blue) (GSE182434). (B) Violin plots comparing the expression levels of DLBCL-defining markers (MS4A1, CD19, CD79A, BCL6, BCL2, MYC, and MKI67) between tumor and normal B cells.


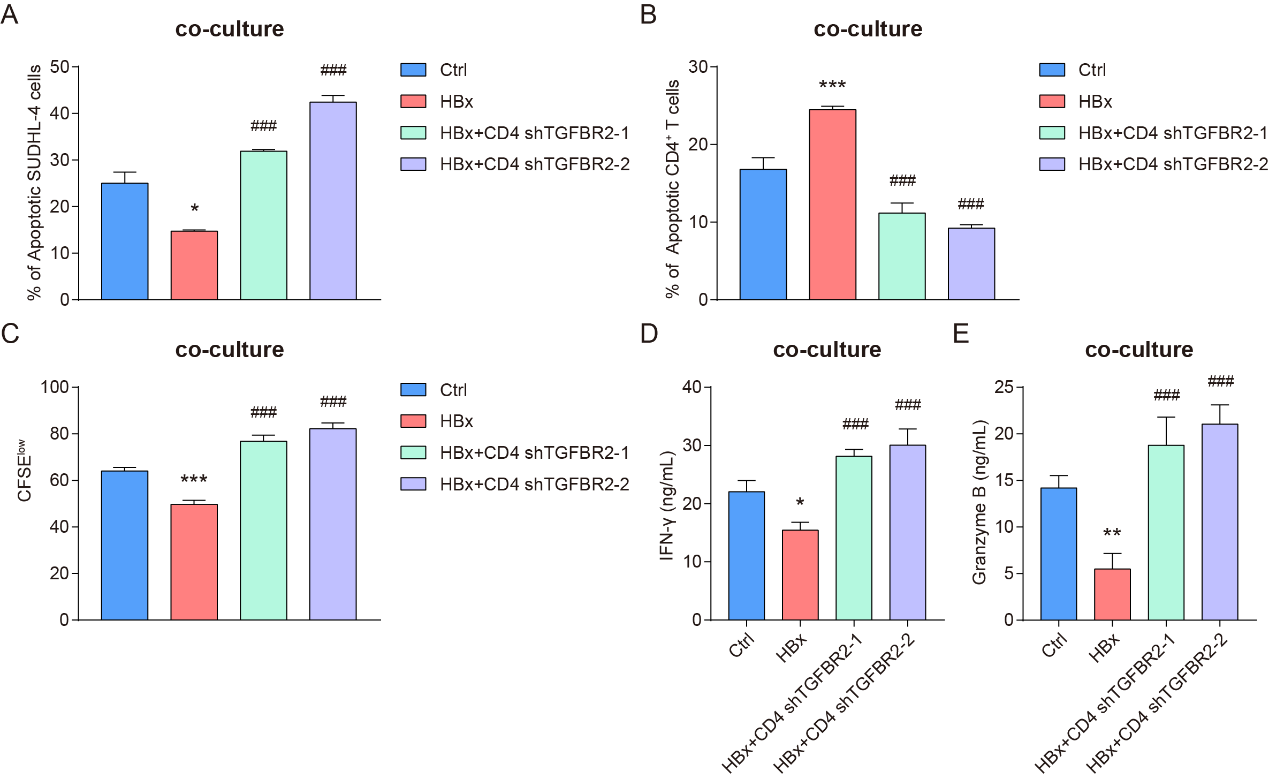


**Supplementary Figure 8. Knockdown of TGFBR2 potentiates the anti-tumor activity of CD4⁺ T cells against HBx-overexpressing DLBCLs.**

(A) Flow cytometry analysis of apoptotic SUDHL-4 cells in the co-culture system. (B, C) Flow cytometry analysis of apoptotic (B) and proliferative (C) CD4⁺ T cells. (D, E) ELISA measurement of IFN-γ (D) and Granzyme B (E) levels in the co-culture supernatant. The data represent mean ± SD of three independent experiments. **p* < 0.05, ***p* < 0.01, and ***/###*p* < 0.001.


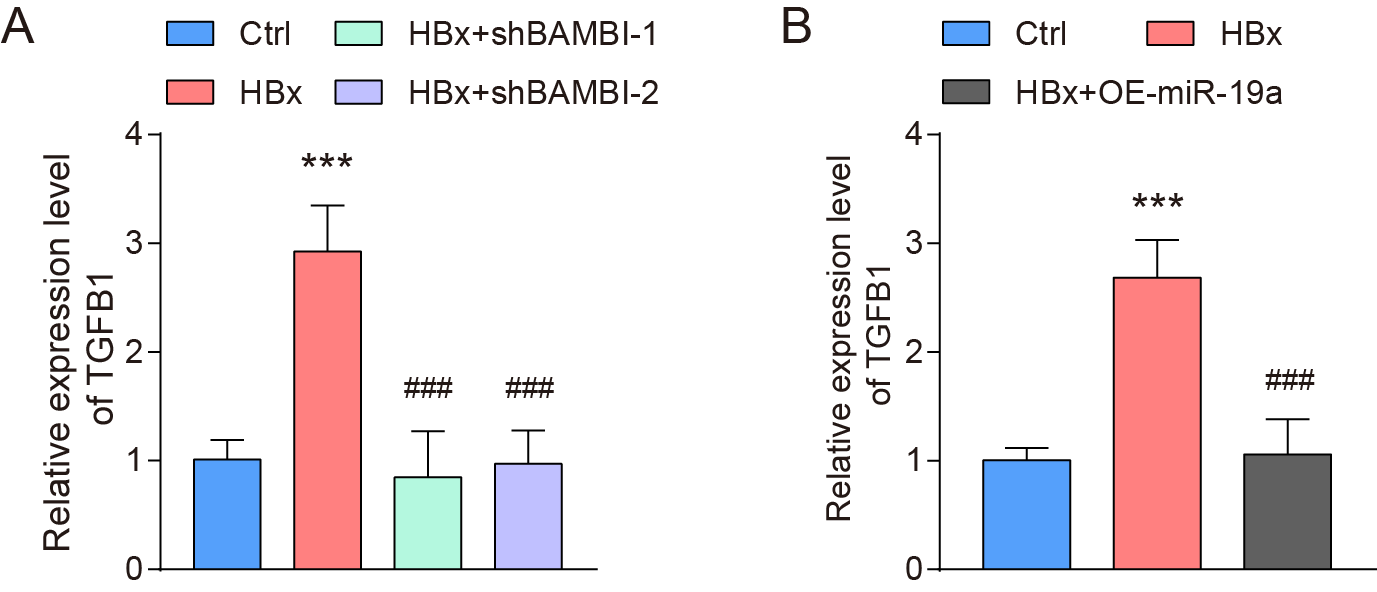


**Supplementary Figure 9. The expression of TGFB1 in SUDHL-4 cells.**

(A, B) The expression of TGFB1 was analyzed by qRT-PCR in the HBx-overexpressing SUDHL-4 cells with knockdown of BAMBI (A) or overexpression of miR-19a (B). The data represent mean ± SD of three independent experiments. ***/###*p* < 0.001.


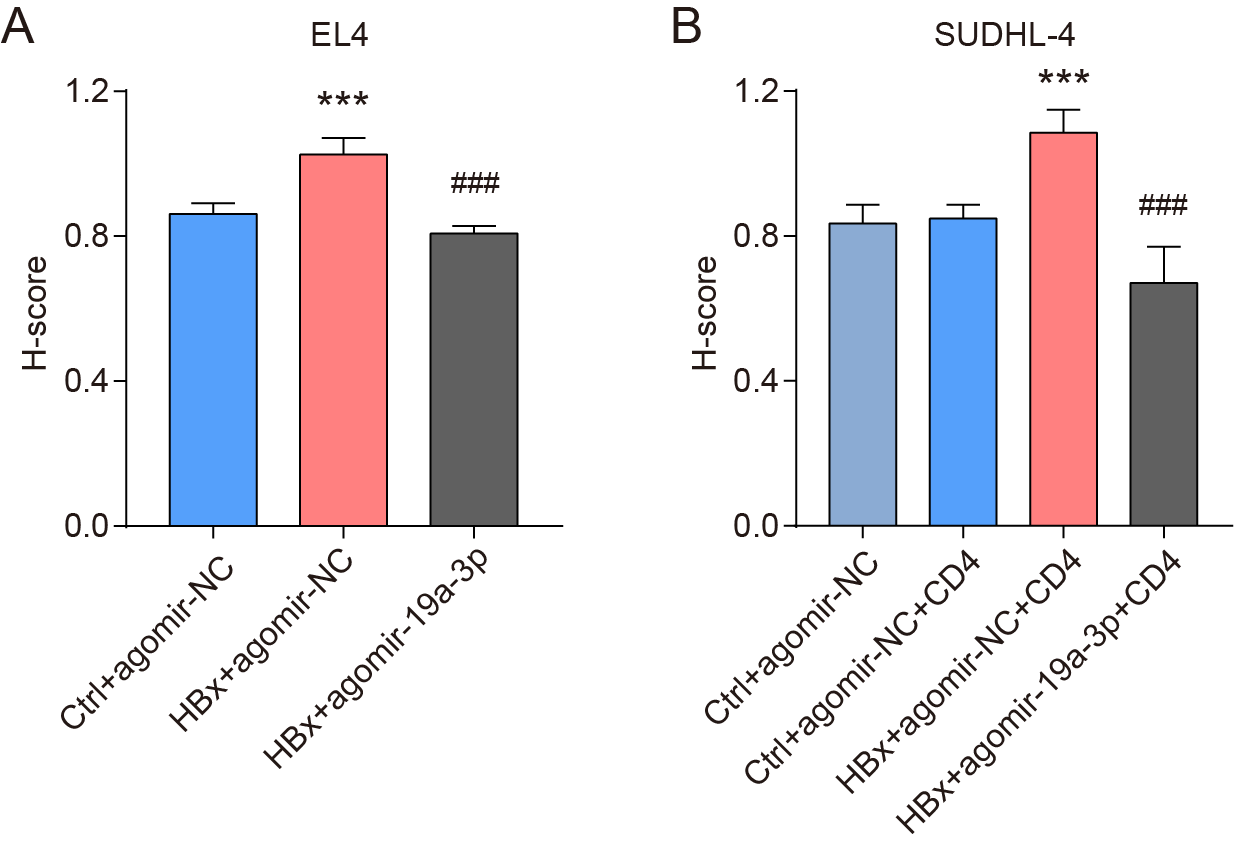


**Supplementary Figure 10. The expression of BAMBI in EL4 and SUDHL-4 tumor tissues.**

(A, B) IHC analysis of BAMBI in the EL4 (A) and SUDHL-4 (B) tumor tissues (n=6 for each group). The error bars represented the mean ± SD.***/###*p* < 0.001.


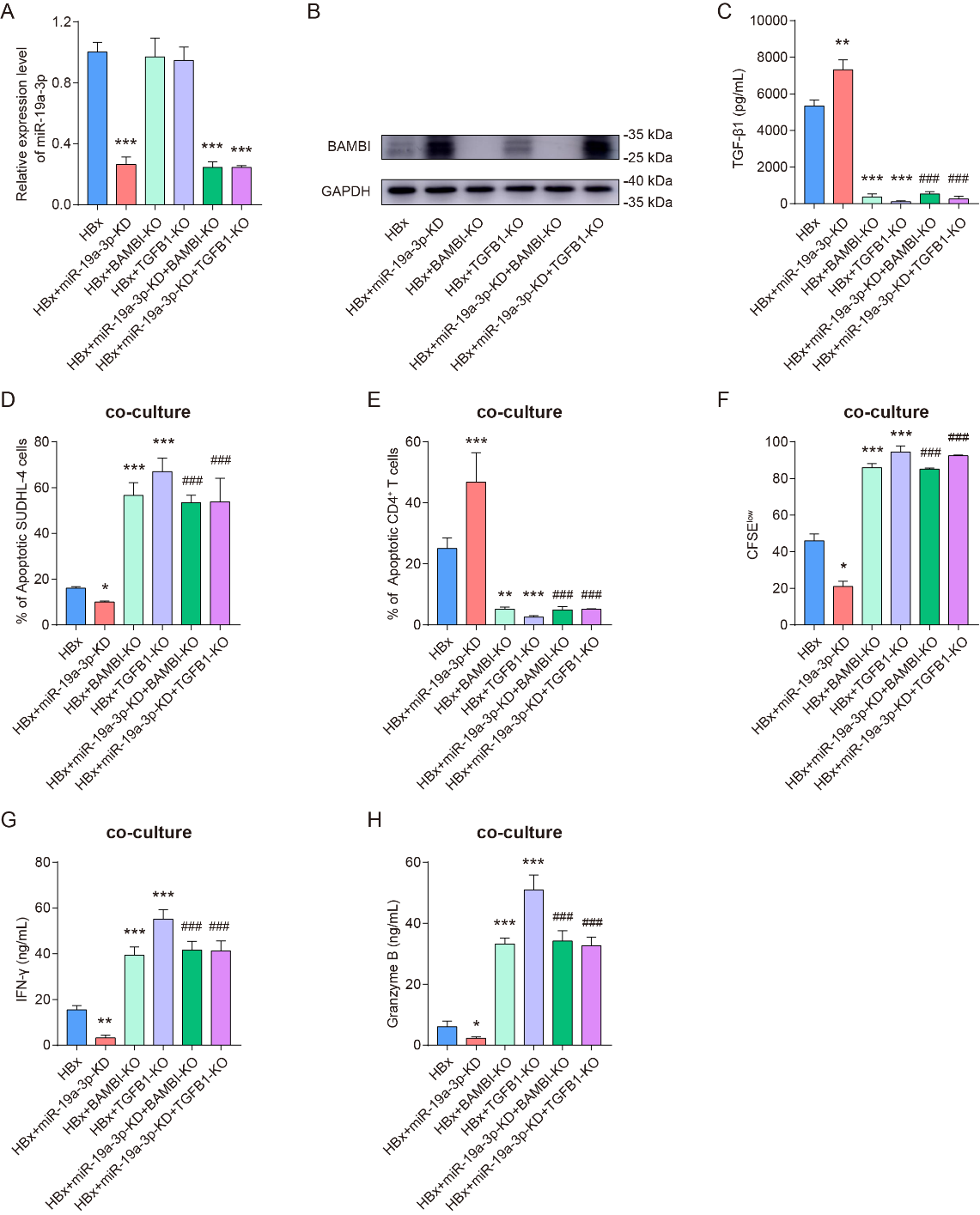


**Supplementary Figure 11. The CRISPR-based evaluation of HBx/miR-19a-3p/BAMBI/TGFB1 axis on CD4^+^ T cell activity.**

(A-C) Validation of target perturbations in six genetically defined SUDHL-4 cell groups: the CRISPR/Cas13d-mediated miR-19a-3p knockdown (miR-19a-3p-KD) was confirmed by qPCR (A), the CRISPR/Cas9-mediated BAMBI and TGFB1 knockout (BAMBI-KO and TGFB1-KO) were validated by Western blot (B) and ELISA (C), respectively. (D) Flow cytometry analysis of apoptotic SUDHL-4 cells in the co-culture system. (E, F) Flow cytometry analysis of apoptotic (E) and proliferative (F) CD4^+^ T cells. (G, H) The ELISA measurement of IFN-γ (G) and Granzyme B (H) levels in the co-culture supernatant. The data represent mean ± SD of three independent experiments. *p < 0.05, **p < 0.01, ***p < 0.001.


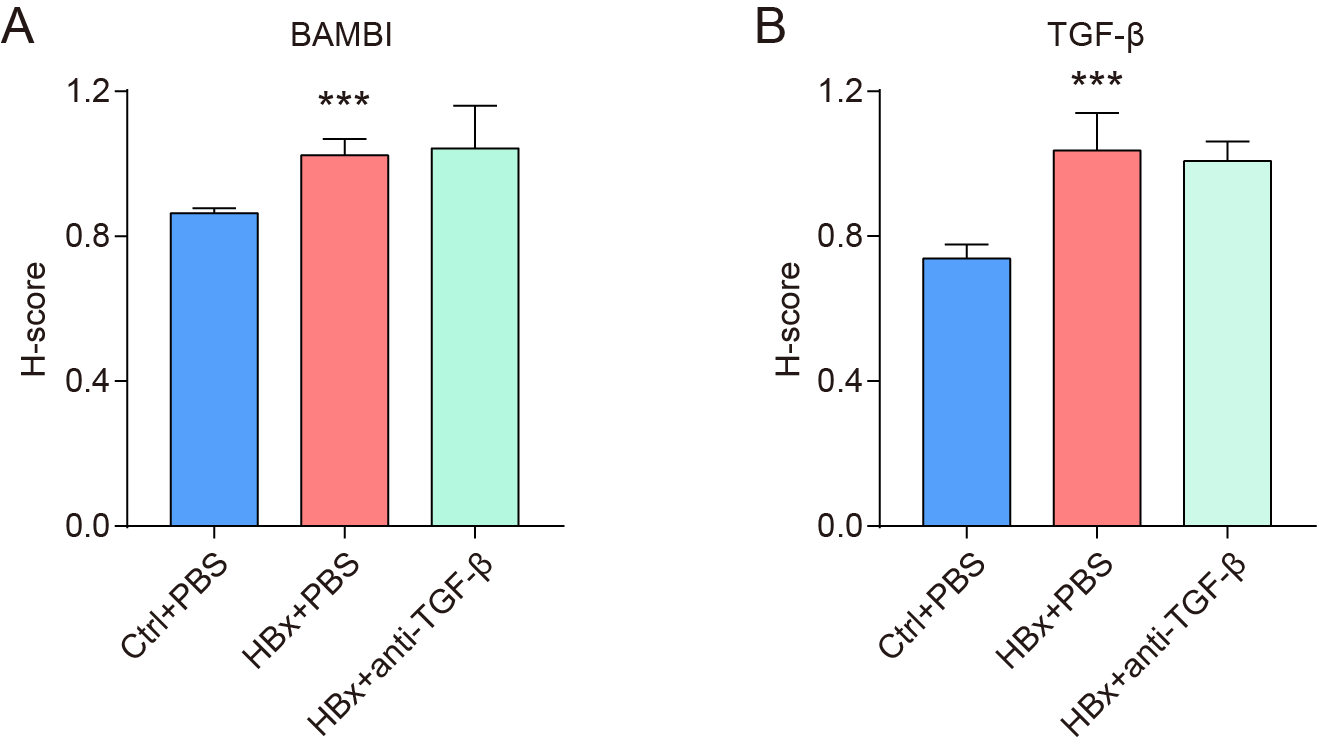


**Supplementary Figure 12. The expression of BAMBI and TGF-β1 in EL4 tumor tissues.**

(A) IHC analysis of BAMBI (A) and TGF-β1(B) in the EL4 tumor tissues with treatment of TGF-β antibody (n=6 for each group). The error bars represented the mean ± SD. ****p* < 0.001.


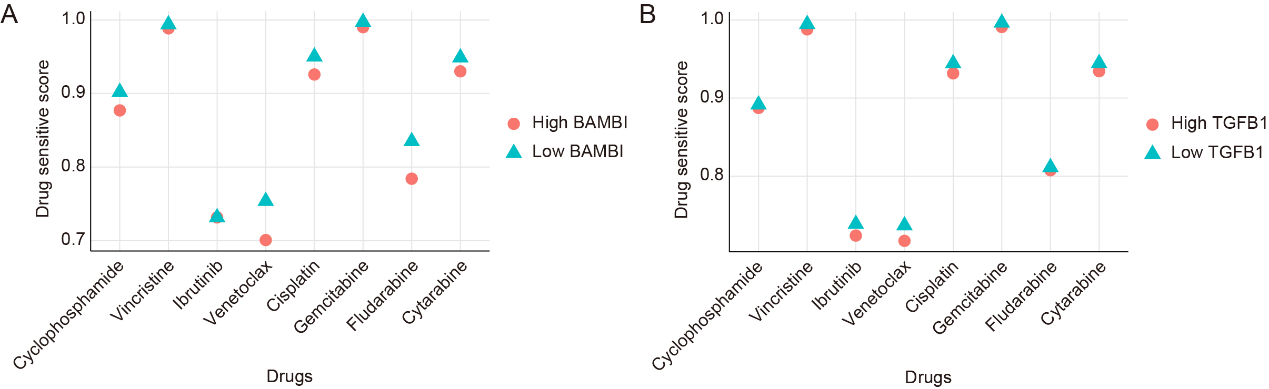


**Supplementary Figure 13. Predicted drug sensitivity in DLBCL samples stratified by BAMBI or TGFB1 expression.**

(A, B) OncoPredict-based drug response scores grouped by BAMBI (A) and TGFB1 (B) expression (GSE125966).


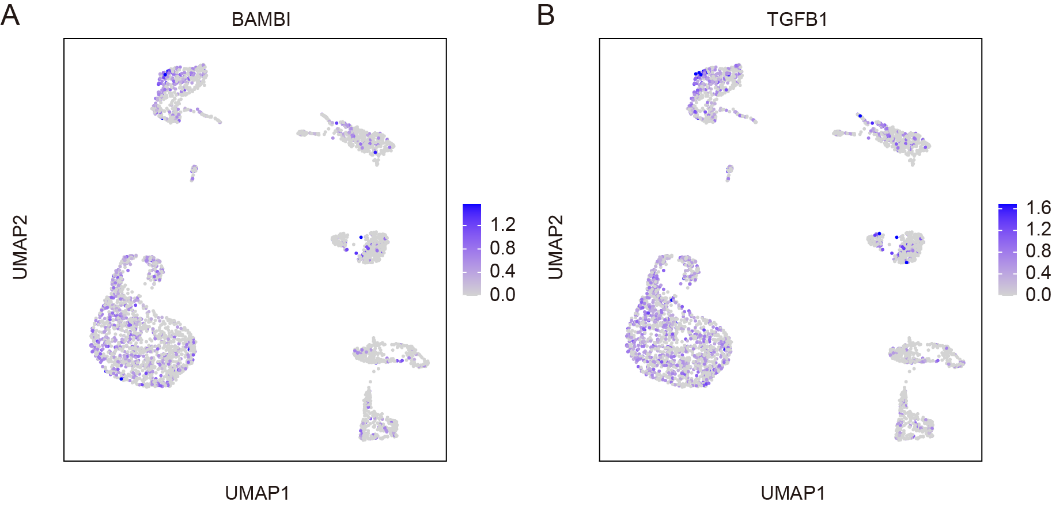


**Supplementary Figure 14. Expression of BAMBI and TGFB1 in malignant B cells at the single-cell level.**

(A, B) UMAP visualization of B cells showing the expression patterns of BAMBI (A) and TGFB1 (B) (GSE182434).
